# Postoperative Delirium Across the Perioperative Pathway: A Structured Narrative Review of Clinical Maturity and Implementation Priorities

**DOI:** 10.3390/diagnostics16213518

**Published:** 2026-10-27

**Authors:** Orsolya Brigitta Katona, Leonard Azamfirei, Mircea Stoian, Lorand Tibor Reman, Alexandra Lazar

**Affiliations:** 1 Institution Organizing University Doctoral Studies (IOSUD), George Emil Palade University of Medicine, Pharmacy, Science, and Technology of Târgu Mureș, 540142 Târgu Mureș, Romania; katona.brigics@gmail.com; 2 Department of Anesthesiology and Intensive Care, George Emil Palade University of Medicine, Pharmacy, Science, and Technology of Târgu Mureș, 540142 Târgu Mureș, Romania; leonard.azamfirei@umfst.ro (L.A.); mircea.stoian@umfst.ro (M.S.); alexandra.lazar@umfst.ro (A.L.); 3 Department of Anesthesiology and Intensive Care, County Emergency Clinic Hospital of Târgu Mureș, 540136 Târgu Mureș, Romania; 4 Department of Anesthesiology and Intensive Care, Clinical County Hospital of Târgu Mureș, 540072 Târgu Mureș, Romania; 5 Department of Urology, George Emil Palade University of Medicine, Pharmacy, Science, and Technology of Târgu Mureș, 540142 Târgu Mureș, Romania; 6 Department of Urology, Clinical County Hospital of Târgu Mureș, 540072 Târgu Mureș, Romania

**Keywords:** postoperative delirium, perioperative care, neuroinflammation, risk factors, prevention, anesthesia, biomarkers

## Abstract

Postoperative delirium (POD) is a highly prevalent but frequently underrecognized complication that disproportionately affects frail and older surgical patients. This structured narrative review aims to synthesize contemporary evidence on adult postoperative delirium across the perioperative pathway and to classify major preventive and therapeutic strategies according to clinical maturity, target population, and implementation readiness. A structured literature search of PubMed/MEDLINE, Scopus, Cochrane Central Register of Controlled Trials (CENTRAL) and Web of Science Core Collection was performed, supplemented by ClinicalTrials.gov, official ESAIC and ASA resources, reference-list screening, and selected seminal pre-2021 publications. Current evidence supports a multifactorial model in which baseline cognitive and physiological vulnerability interacts with inflammatory, pharmacological, hemodynamic, nociceptive, sleep-related, and environmental stressors. No single anesthetic technique, processed-EEG target, blood-pressure threshold, cerebral oximetry strategy, or pharmacological agent consistently prevents POD across surgical populations. Multicomponent non-pharmacological prevention has the most consistent support, whereas dexmedetomidine and other pharmacological approaches appear context-dependent, and biomarkers and metabolic targets remain investigational. Future progress will depend on standardized delirium ascertainment, better phenotyping, multimodal prediction and prevention strategies, and reliable implementation systems that identify vulnerability early and maintain effective preventive care across transitions.

## 1. Introduction

Postoperative delirium (POD) is an acute and fluctuating neurocognitive syndrome that typically emerges during the early postoperative period and reflects the interaction between pre-existing cerebral vulnerability and perioperative physiological stress. Despite being one of the most frequent neurocognitive complications of surgery in adults, POD remains substantially underrecognized, particularly when it presents in a hypoactive form. It should no longer be regarded as a transient or benign consequence of anesthesia, as accumulating evidence links delirium with prolonged hospitalization, functional decline, persistent cognitive impairment, increased risk of subsequent dementia, and higher long-term mortality. These associations are especially important in older and frail surgical populations, in whom even relatively modest perioperative disturbances may exceed the brain’s capacity to maintain stable and coordinated neural activity. As the surgical population continues to age, prevention has therefore become a major focus of perioperative research. Contemporary strategies increasingly emphasize multicomponent non-pharmacological interventions and early identification of modifiable risk factors, while efforts to establish reliable biomarkers, individualized monitoring approaches, and effective pharmacological prophylaxis continue to produce heterogeneous and, in some areas, conflicting results [1,2,3,4,5,6].

Postoperative delirium should be distinguished from other perioperative neurocognitive disorders. According to the 2018 consensus nomenclature, cognitive decline identified within 30 days after surgery is termed delayed neurocognitive recovery, whereas postoperative neurocognitive disorder refers to cognitive decline identified from 30 days to 12 months after surgery; these entities require appropriate cognitive and functional assessment and should not be diagnosed using a delirium screening instrument alone [7]. Postoperative delirium instead represents an acute, fluctuating disturbance of attention, awareness, and cognition occurring in the postoperative period [7,8]. Emergence of delirium or agitation occurring during or immediately after recovery from anesthesia should also be distinguished from POD developing later in the postoperative course [9].

Postoperative delirium is increasingly recognized as a heterogeneous clinical syndrome rather than a uniform manifestation of postoperative cognitive dysfunction. Instead, the syndrome is now rigorously stratified into distinct phenotypes based on motoric activity, temporal onset, and clinical severity. This distinction is particularly relevant because hypoactive, hyperactive, and mixed forms may differ substantially in their clinical visibility and immediate care requirements, while emergence-related and delayed postoperative presentations may arise within different perioperative contexts. Phenotypic characterization should therefore be viewed as clinically relevant rather than purely descriptive, as it can improve recognition, guide bedside assessment, and support more appropriate interpretation of prognosis and subsequent neurocognitive outcomes [1,3,10].

At the bedside, differentiation of motoric delirium subtypes should begin with a structured assessment of arousal. Instruments such as the Richmond Agitation–Sedation Scale (RASS) help establish the patient’s level of consciousness before more specific delirium screening tools are applied, thereby reducing the risk that hypoactive presentations are misinterpreted as expected postoperative fatigue [11,12]. Hyperactive delirium is usually the most clinically apparent form, presenting with agitation, restlessness, aggression, or combativeness; however, it represents only a minority of cases [2,10]. By contrast, hypoactive delirium is often subtle and therefore frequently overlooked, despite its association with substantial morbidity and increased six-month mortality. These patients may appear quiet, lethargic, withdrawn, or unusually somnolent [3,10]. A third pattern, mixed delirium, is characterized by fluctuation between hyperactive and hypoactive features, often within the same 24 h period [10,13].

From a temporal perspective, postoperative delirium may present either during emergence from anesthesia or later in the postoperative course. Emergence delirium occurs during the transition from anesthesia to wakefulness and is often short-lived; however, in some patients, it may represent an early manifestation of vulnerability and precede delayed-onset postoperative delirium. The latter typically develops between postoperative days 1 and 5 and appears to be more closely associated with sleep disruption, circadian dysregulation, and systemic inflammatory responses. Beyond timing, delirium can also be characterized according to severity. Increasing evidence suggests that the intensity and duration of delirium symptoms may reflect the magnitude of underlying neurovascular and network dysfunction.

Recent reviews from Ding et al. and Smirnov et al. have substantially advanced the conceptual and clinical framework surrounding postoperative delirium and the broader spectrum of perioperative neurocognitive disorders [14,15]. Dilmen et al. provided a clinically oriented guide to postoperative neurocognitive disorders, emphasizing identification of vulnerable patients, individualized perioperative risk reduction, and the Safe Brain Initiative as a multidisciplinary approach integrating preoperative assessment, appropriate anesthetic depth, analgesia, hemodynamic stability, and non-pharmacological preventive measures across the perioperative pathway [16]. Ahmadzadeh et al. subsequently focused more specifically on postoperative delirium, integrating current concepts of multifactorial pathogenesis with clinically relevant predisposing and precipitating factors, validated approaches to delirium detection, multicomponent non-pharmacological prevention, and the evolving role of pharmacological strategies. Their review further highlighted persistent uncertainties regarding optimal hemodynamic targets, electroencephalography-guided anesthetic titration, biomarkers of perioperative neuroinflammation, and the development of more individualized risk-stratified prevention strategies [17]. Paternò et al. considered postoperative delirium within the wider framework of perioperative neurocognitive disorders, synthesizing contemporary evidence on neuroinflammation, blood–brain barrier dysfunction, cerebral perfusion, neurotransmitter alterations, patient vulnerability, preventive strategies, pharmacological interventions, and emerging biomarkers. This review illustrates both the increasing breadth of mechanistic and therapeutic research in this field and the continuing uncertainty surrounding the translation of several promising interventions into routine clinical practice [18].

Collectively, these contributions demonstrate a progressive shift from viewing postoperative delirium as an isolated postoperative complication toward understanding it as part of a multidimensional perioperative neurocognitive continuum requiring coordinated risk recognition, prevention, detection, and management. The present review builds on this evolving literature while adopting a complementary implementation-oriented perspective: rather than considering the available interventions solely according to biological plausibility or reported efficacy, it examines their applicability across the perioperative pathway and explicitly distinguishes strategies supported for routine clinical practice from those appropriate only in selected clinical contexts and those that remain investigational. By linking evidence interpretation with clinical maturity, population-specific applicability, safety considerations, and implementation requirements, this framework is intended to clarify not only which strategies have been studied but also where, for whom, and under what conditions the available evidence currently supports their use.

## 2. Materials and Methods

This article is a structured narrative review designed to integrate evidence across the adult perioperative pathway and support clinically contextualized interpretation. The search and selection procedures are reported transparently, but the review was not designed to produce an exhaustive systematic synthesis or a pooled treatment-effect estimate. A literature search of PubMed/MEDLINE, Scopus, Cochrane Central Register of Controlled Trials (CENTRAL) and Web of Science Core Collection was conducted for English-language publications published between 1 January 2021 and 26 September 2026. Search strategies combined database-specific controlled vocabulary with title- and abstract-based terms related to postoperative, perioperative, postsurgical, and postanesthetic delirium. Database-specific syntax and indexing terms were adapted for each platform. Complete database-specific search strategies, platform details, execution dates, and numbers of records retrieved are provided in Supplementary Table S1.

No age, human-subject, or publication-type filters were applied during the electronic search. Eligibility was intentionally broad to reflect the integrative scope of this structured narrative review. The principal evidence base comprised publications involving adults aged ≥18 years undergoing elective or emergency surgical procedures and addressing postoperative delirium as a principal outcome, secondary outcome, or substantial subject of investigation. Given the multifactorial nature of POD and the purpose of the review to integrate evidence across the perioperative pathway, publications were also considered when they provided directly relevant information on delirium ascertainment and phenotyping, perioperative neurocognitive vulnerability, pathophysiological mechanisms, risk stratification, biomarkers, prehabilitation, physiological or anesthetic management, pharmacological or non-pharmacological prevention, delirium treatment and safety, implementation, or clinically relevant outcomes.

Eligible evidence therefore included randomized controlled trials, prospective and retrospective observational studies, systematic reviews and meta-analyses, narrative reviews with substantive relevance to POD or perioperative neurocognitive disorders, clinical guidelines and consensus statements, and selected clinical or mechanistic studies that contributed directly to interpretation of the above domains. Evidence derived from broader hospitalized or intensive-care populations, or from perioperative neurocognitive outcomes other than delirium, could be retained when it provided clinically relevant contextual information concerning mechanisms, safety, treatment, implementation, or emerging therapeutic approaches. Such evidence was interpreted as contextual or indirect evidence and was not treated as equivalent to direct evidence of POD prevention or treatment efficacy.

Publications were excluded when they focused exclusively on pediatric emergence delirium, had no substantive relationship to perioperative delirium or the predefined clinical domains, consisted of case reports, editorials or letters without substantive evidence, were available only as conference abstracts without a peer-reviewed full-text publication, or were not published in English. The principal eligibility criteria are summarized in Table 1.

Supplementary searches were undertaken in ClinicalTrials.gov and through official resources of the European Society of Anesthesiology and Intensive Care (ESAIC) and the American Society of Anesthesiologists (ASA). Reference lists of relevant reviews, guidelines, and key primary studies were additionally examined to identify important publications not captured by the primary database search. The 2021–2026 publication window governed eligibility for records retrieved by the primary database search, not the publication dates of every primary study represented in a recent systematic review or meta-analysis. Evidence syntheses published within this window were eligible even when they included pre-2021 trials or cohorts. Older primary reports were cited separately only when necessary to explain a foundational definition, validated instrument, landmark trial, or enduring clinical concept, or when the original report was needed to interpret a specific finding. These exceptions were selected purposively and were not treated as part of the time-limited database cohort.

Because the purpose of this review was to provide an integrative and clinically oriented interpretation of the literature rather than an exhaustive systematic review or quantitative estimate of treatment effects, no meta-analysis or formal risk-of-bias or GRADE assessment was performed. Evidence was synthesized narratively according to predefined clinical domains spanning the perioperative pathway. Greater interpretive weight was given to contemporary clinical guidelines, systematic reviews and meta-analyses, randomized controlled trials, large observational studies, and studies providing clinically or mechanistically distinct information. For each major clinical question, conclusions from evidence syntheses were interpreted alongside, rather than added numerically to, primary reports already contained within those syntheses. During revision, a standardized evidence-extraction and traceability map was created to verify principal quantitative statements, the directness and limitations of pivotal evidence, and the clinical-maturity classifications. This revision-stage evidence map is provided in Supplementary Table S2. The revision-stage evidence extraction and source verification were performed by OBK and checked by LTR.

Findings were interpreted according to study design, consistency across studies, directness of evidence, relevance of the investigated population and outcome, safety considerations, and clinical applicability. Particular attention was given to discrepancies arising from differences in delirium definitions, assessment instruments, surveillance periods, surgical populations, intervention protocols, and perioperative settings, because these factors represent important sources of heterogeneity within the POD literature.

To facilitate translation of the evidence into clinical practice, preventive and therapeutic strategies were grouped according to their current level of clinical maturity: (1) strategies supported for routine clinical practice; (2) strategies that may be considered in selected populations or clinical contexts; and (3) investigational approaches requiring further validation. Classification was based on the overall consistency and directness of the available evidence, replication across clinical settings, safety profile, feasibility of implementation, and concordance with contemporary clinical guidelines. These categories were intended as a pragmatic framework for clinical interpretation and should not be considered equivalent to formal GRADE certainty ratings. Study selection was undertaken by the authors using an intentionally inclusive approach appropriate to the broad clinical scope of a structured narrative review. Title/abstract screening was performed by OBK, with uncertain records retained for full-text assessment; ambiguous full-text eligibility decisions were reviewed with LTR and resolved by consensus. Full-text selection considered not only strict population–outcome matching but also the contribution of each publication to the interpretation of POD diagnosis, mechanisms, vulnerability, prevention, treatment, safety, implementation, or perioperative neurocognitive context. Final inclusion was determined by author consensus on clinical relevance, methodological contribution, and the extent to which a publication added distinct information to the narrative synthesis. This approach was deliberately inclusive rather than exhaustive or quantitatively restrictive and was consistent with the narrative-review methodology and reporting principles reflected in the Scale for the Assessment of Narrative Review Articles (SANRA) [19]. Reference management and duplicate removal were performed using Zotero 10.0.6 (64-bit). In keeping with the narrative-review design, the search was structured and transparently documented, while study selection remained deliberately inclusive and clinically oriented rather than being restricted to a narrowly defined systematic-review question. The identification and selection process is presented in Supplementary Figure S1, in a PRISMA-style flow diagram solely to improve transparency and reproducibility of reporting. No prospective review protocol was registered. The review was conducted as a structured narrative synthesis. 

## 3. Narrative Synthesis

### 3.1. Delirium Ascertainment: A Major Source of Clinical and Research Heterogeneity

Postoperative delirium is inherently difficult to measure because its defining features—fluctuation, inattention, altered arousal, and cognitive disturbance—may vary substantially over the course of a single day. Consequently, reported incidence is influenced not only by patient and surgical characteristics, but also by the method and intensity of delirium ascertainment. Differences in screening instruments, timing and frequency of assessment, postoperative surveillance windows, assessor training, and the handling of reduced arousal can all contribute to heterogeneity across studies. This is particularly relevant for hypoactive delirium, which may be misclassified as fatigue, residual anesthetic effect, or expected postoperative somnolence unless arousal is assessed systematically before cognitive screening. Instruments such as the Richmond Agitation–Sedation Scale can therefore complement delirium-specific tools by clarifying whether altered consciousness may interfere with subsequent assessment [11,12].

Validated instruments including the 4AT, CAM, and CAM-ICU provide structured approaches to delirium detection, but they are not interchangeable and were developed for somewhat different clinical environments. Their performance in routine practice also depends on whether assessments are completed consistently and repeated when clinical status changes. Large-scale implementation data demonstrate that structured screening can identify patients with substantially worse outcomes, while contemporary studies show considerable variation in completion rates and positive-screen prevalence when tools are deployed across routine hospital systems [20,21]. A negative assessment at a single time point therefore cannot reliably exclude a fluctuating syndrome.

These differences have important implications for interpretation of the POD literature. Apparent variation in incidence or intervention efficacy may reflect, at least in part, differences in ascertainment rather than true biological or therapeutic effects. Standardized instruments, predefined surveillance periods, repeated assessment, and transparent reporting of delirium methodology should therefore be considered essential elements of future perioperative trials.

### 3.2. Pathophysiology and Interacting Mechanisms

Postoperative delirium is no longer considered a single localized event, but rather a complex systemic-to-neuronal failure. Three interacting mechanistic domains of POD highlighted in this review are further discussed in this section [2,5,22].

#### 3.2.1. The Neuroinflammatory Cascade

At the core of postoperative delirium is the concept of surgically induced systemic inflammation translating into neuroinflammation [23]. Surgical trauma may trigger the release of Damage-Associated Molecular Patterns (DAMPs) from the local tissue injury into the circulation, activating the innate immune system. This later leads to an elevation of pro-inflammatory cytokines, especially interleukin-1β (IL-1β) and tumor necrosis factor-α (TNFα). In the frail elderly brain, these cytokines precipitate endothelial dysfunction and degradation of tight junction proteins, leading to a compromised blood–brain barrier (BBB) [22,24,25]. Peripheral signals activate the microglia, considered the brain’s immune cells, which shift to a pro-inflammatory phenotype, releasing reactive oxygen species (ROS) and additional cytokines that induce synaptic remodeling, leading to neuronal injury. Peripheral immune cells can infiltrate the brain parenchyma, aggravating the neuroinflammation [24,25,26].

#### 3.2.2. Neurotransmitter Imbalance

Neurotransmitter dysregulation is a key component of contemporary models of postoperative delirium (POD), but is increasingly regarded as one element of a broader pathophysiological network involving neuroinflammation, impaired cerebral homeostasis, disrupted large-scale neural connectivity, and circadian dysregulation. Current evidence suggests that POD reflects a dynamic imbalance among cholinergic, dopaminergic, GABAergic, glutamatergic, and adrenergic systems, with the contribution of each pathway varying according to baseline cerebral vulnerability and the magnitude of perioperative stress [27,28].

- Cholinergic deficiency: Acetylcholine is fundamental to attention, memory, learning, and modulation of inflammatory responses. Age-related reductions in cholinergic reserve increase susceptibility to surgical stress, systemic inflammation, cerebral hypoperfusion, and anticholinergic medications [27,28]. In a prospective cohort enrolling 421 older surgical patients, Moellmann et al. reported available anticholinergic burden (ACB) scores for 413 participants. Increased preoperative anticholinergic burden, defined as an ACB score ≥ 3, was associated with higher odds of POD (OR 2.79, 95% CI 1.31–5.97), with reported incidences of 25.6% versus 11.0% among patients with and without increased burden, respectively [29]. Although consistent with an association between anticholinergic exposure and delirium susceptibility, these findings should be interpreted cautiously because of the observational design, potential residual confounding, and limited generalizability beyond the studied population. They do not establish cholinergic dysfunction as a causal mechanism or demonstrate that reducing anticholinergic burden prevents POD [27,29].
- Dopaminergic and noradrenergic dysregulation: Alterations in dopaminergic and noradrenergic signaling have been implicated in delirium, potentially interacting with cholinergic dysfunction, neuroinflammation, and physiological stress. However, direct biochemical evidence is limited and heterogeneous, and recent data do not consistently support a simple model of dopaminergic excess [30].
- GABAergic and adrenergic dysregulation: Altered inhibitory GABAergic signaling and excessive noradrenergic activity may further destabilize cortical network function. Dexmedetomidine reduces central norepinephrine release while providing an arousable, sleep-like sedation, and its apparent protective effect likely reflects combined adrenergic modulation, opioid-sparing, sleep-preserving, and anti-inflammatory actions [31,32]. Clinical evidence regarding dexmedetomidine is discussed in Section 3.5.4. These findings indicate that POD is better understood as a disorder of disrupted neurochemical homeostasis than of a single neurotransmitter abnormality, with cholinergic insufficiency, dopaminergic and noradrenergic overactivity, and altered inhibitory signaling interacting with inflammation and cerebral vulnerability to produce the characteristic fluctuating syndrome of delirium [14].

#### 3.2.3. Brain Network Connectivity and Neural Dysfunction

**Figure 1:**
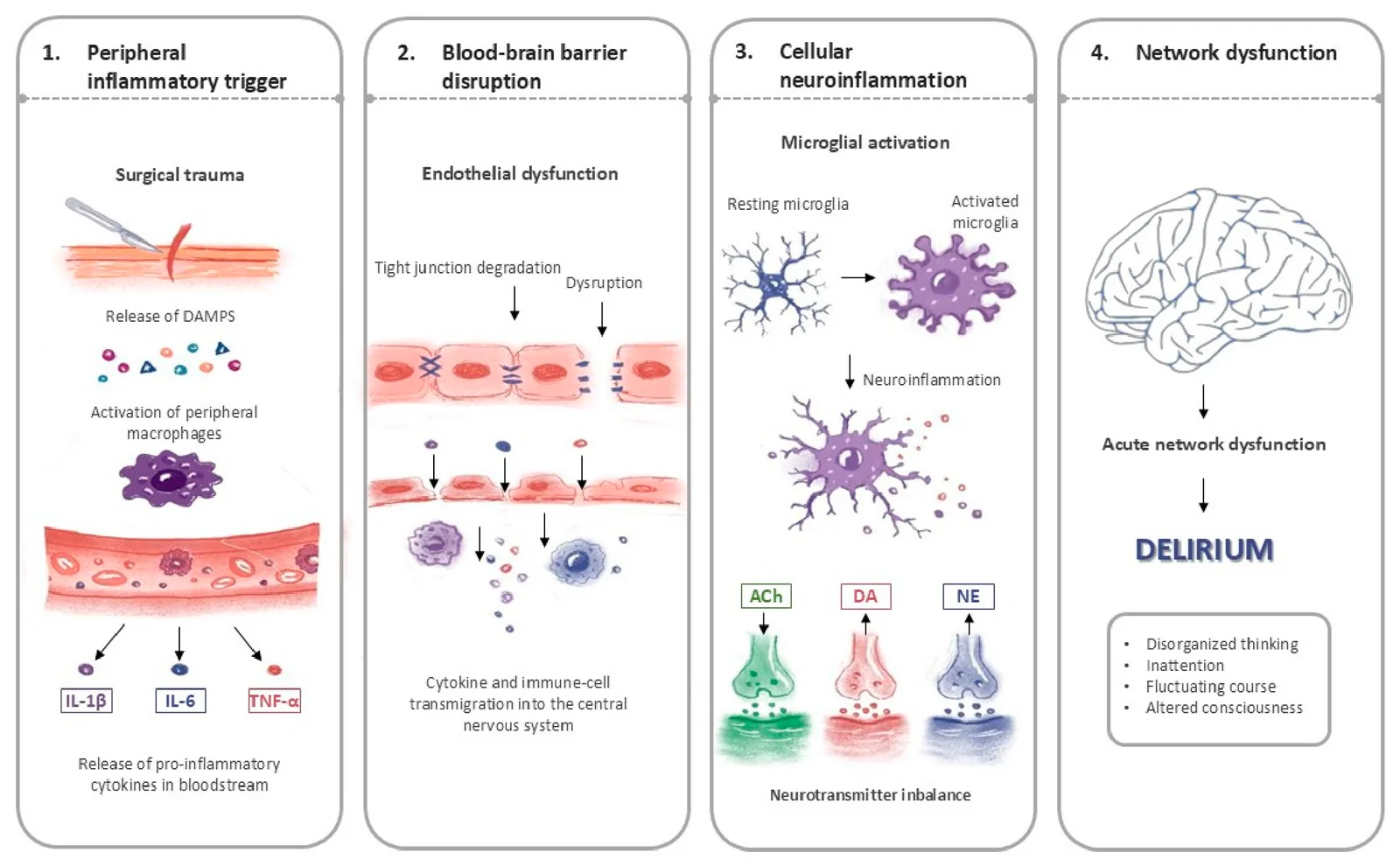
Proposed interacting mechanisms linking surgical stress to postoperative delirium.

Postoperative delirium is increasingly conceptualized as a disorder of large-scale brain network integrity rather than the consequence of dysfunction within a single anatomical “delirium center.” In this model, acute perioperative stressors act on a vulnerable brain and disrupt the dynamic coordination between networks responsible for arousal, attention, internal awareness, and executive control. The default mode network, particularly its medial prefrontal and posterior cingulate–precuneus hubs, is relevant because of its role in self-referential processing and baseline conscious integration, while frontoparietal and salience networks appear critical for externally directed attention, cognitive flexibility, and the switching between internal and environmental demands. Disruption of communication across these systems may therefore provide a plausible neurobiological substrate for the fluctuating awareness, inattention, disorientation, and impaired executive function that define POD. Contemporary electrophysiological data further support this network-based interpretation: delirium is commonly associated with generalized EEG slowing, characterized by attenuation of organized alpha activity and a relative shift toward theta and delta frequencies. Rather than representing a simple marker of reduced cerebral metabolism, these EEG changes are better interpreted as a real-time signature of impaired cortical integration, reduced neural complexity, and failure of efficient information transfer across distributed cortical–subcortical circuits. Current evidence therefore supports a threshold model in which POD emerges when pre-existing vulnerability—such as advanced age, frailty, cognitive impairment, neurodegenerative burden, or impaired cerebral reserve—is exposed to perioperative triggers including inflammation, sleep disruption, anesthetic exposure, nociceptive stress, and hemodynamic instability. Within this framework, delirium reflects a transient but clinically meaningful collapse of network homeostasis, in which the brain can no longer maintain coordinated arousal and cognitive integration under the burden of surgical inflammatory and physiological stress [33,34,35,36]. These pathways, also represented in Figure 1, should be regarded as interacting and partially overlapping mechanisms.

### 3.3. Preoperative Risk Stratification and Patient Optimization

Effectively reducing the incidence of postoperative delirium (POD) requires a fundamental shift in clinical practice from reactive postoperative management to proactive preoperative risk stratification and individualized optimization. Systematic evaluation of a patient’s baseline neurological resilience in relation to the anticipated physiological stress of surgery enables clinicians to identify vulnerability profiles and implement targeted preventive interventions before surgical stress response occurs [37].

#### 3.3.1. The Vulnerable Brain and Pre-Existing Frailty

Contemporary risk stratification for postoperative delirium is increasingly based on the concept of the “vulnerable brain.” Within a stress–vulnerability model, POD develops when the cumulative burden of perioperative insults exceeds an individual’s capacity to maintain cerebral and systemic homeostasis. Baseline susceptibility is influenced by cognitive impairment, frailty, multimorbidity, sensory dysfunction, and functional dependence, whereas precipitating factors include surgical trauma, inflammation, pain, metabolic disturbance, sleep disruption, hemodynamic instability, and deliriogenic medications. Patients with substantial pre-existing vulnerability may therefore develop delirium after relatively modest physiological stress, while those with greater reserve generally require more severe or sustained insults before the clinical threshold is crossed [8,33,34,35,38].

Frailty is particularly relevant because it reflects a multidimensional reduction in physiological reserve and impaired recovery from acute stress. It should not be considered a direct surrogate for structural brain aging, but rather a clinical marker of reduced resilience across interacting systems. Frail patients commonly exhibit multimorbidity, diminished functional reserve, altered autonomic regulation, and chronic low-grade inflammation, all of which may amplify the cerebral consequences of surgery. Physical and cognitive vulnerability frequently coexist and may have additive effects on delirium risk. Accordingly, preoperative assessment should extend beyond chronological age and conventional comorbidity measures to include structured frailty and cognitive screening. The Clinical Frailty Scale offers a pragmatic option for routine practice, whereas the Fried Frailty Phenotype provides a more detailed assessment when time and resources permit. Identifying frailty should trigger evaluation of modifiable vulnerabilities and implementation of individualized, multicomponent preventive strategies rather than serve as an endpoint in itself. Cognitive vulnerability represents a related but distinct dimension of risk. Cognitive reserve describes the capacity to maintain function despite age-related or pathological brain changes and may explain why patients with similar cerebral pathology respond differently to perioperative stress. It is influenced by lifelong educational, occupational, intellectual, and social factors but should not be equated with baseline cognitive performance. Pre-existing cognitive impairment remains one of the most consistent predictors of POD and is frequently underrecognized before surgery [8,34,35,38,39,40,41].

Brief screening tools such as the Mini-Cog can be incorporated into time-limited pathways, while the MoCA provides a broader assessment of attention, executive function, memory, language, and visuospatial ability. These instruments do not directly measure cognitive reserve; rather, they identify deficits that may indicate reduced cerebral resilience. Abnormal findings should support individualized planning, medication review, avoidance of excessive sedation, sensory optimization, structured delirium surveillance, and early multicomponent prevention [8,34,35,38]. A comprehensive stratification of both predisposing and precipitating risk factors is detailed in Table 2.

#### 3.3.2. Novel Blood-Based Biomarkers

Blood-based biomarkers have emerged as promising tools for improving risk stratification and understanding the biological mechanisms underlying postoperative delirium (POD). Among these, plasma neurofilament light chain (NfL) has attracted particular interest because it reflects neuroaxonal injury and may identify cerebral vulnerability that is not fully captured by age, comorbidity, or cognitive assessment. Elevated preoperative NfL concentrations may indicate subclinical neurodegeneration, cerebrovascular disease, or reduced neuronal reserve, whereas postoperative increases may reflect acute perioperative neuronal injury. Tau-related biomarkers provide complementary information, as total and phosphorylated tau are associated with synaptic dysfunction and neurodegenerative processes. However, the temporal relationship between these biomarkers and clinically manifest delirium remains incompletely understood, and neither marker can currently be considered diagnostic. Their principal value lies in biological phenotyping and enrichment of high-risk populations rather than stand-alone clinical decision-making [34,35,42,43].

Inflammatory markers, including interleukin-6 and C-reactive protein, may complement neuronal-injury markers in biological phenotyping, but their perioperative changes are nonspecific. Age, renal function, comorbidity, surgical insult, sampling time, and assay variability further limit interpretation across cohorts. Neither neuronal-injury nor inflammatory markers have validated thresholds for routine diagnosis or biomarker-guided POD prevention. Their incremental predictive value beyond frailty and cognitive assessment, along with feasibility and cost-effectiveness, requires prospective evaluation in diverse surgical populations [34,35,42,43].

#### 3.3.3. Prehabilitation: Brain Training and the Impact of Physical Exercise

Recognizing that both physical frailty and cognitive reserve are malleable targets has driven the emergence of prehabilitation—a multimodal, proactive optimization strategy designed to enhance a patient’s physiological buffer before elective surgery [8,38]. Prehabilitation has emerged as a strategy to increase physiological and cognitive resilience before surgery, particularly in older or otherwise vulnerable patients undergoing major elective procedures. Physical prehabilitation most commonly combines aerobic or high-intensity interval exercise with resistance training, with the primary aim of improving cardiorespiratory fitness, muscular strength, and the capacity to tolerate perioperative physiological stress. Recent randomized trials have demonstrated that meaningful improvements in functional capacity can be achieved within relatively short preoperative intervals, including programs lasting approximately three to four weeks [44,45,46]. In selected high-risk patients undergoing colorectal surgery, a three-week individualized exercise program was associated with a reduction in overall postoperative complications, while short-term high-intensity interval training has also been shown to improve preoperative fitness [44,45]. The biological rationale for a potential neuroprotective effect is plausible: regular exercise may influence neurotrophic signaling, systemic inflammatory activity, vascular function, and metabolic homeostasis. However, these mechanisms have not been established as mediators of postoperative delirium prevention in clinical surgical trials. Accordingly, improvements in physical fitness should not be assumed to translate directly into a lower incidence of POD. Current evidence supports physical prehabilitation as a means of strengthening general physiological reserve, whereas its specific effect on perioperative cerebral resilience remains to be determined [44,45]. This distinction is supported by Feng et al., who reviewed the relationship between perioperative exercise and perioperative neurocognitive disorders. Lower preoperative physical activity, reduced exercise capacity, and diminished skeletal muscle mass have been associated with greater POD risk in several surgical populations, suggesting that physical function may contribute to perioperative vulnerability. Exercise may also influence neuroinflammation, neurotrophic signaling, mitochondrial homeostasis, synaptic plasticity, and systemic metabolic function; however, much of the mechanistic evidence derives from preclinical models, while the available human studies are heterogeneous and frequently evaluate broader postoperative cognitive outcomes rather than POD as a prespecified primary endpoint. Consequently, perioperative exercise is best regarded as a potentially beneficial component of individualized prehabilitation rather than as an established delirium-specific preventive intervention, and adequately powered prospective trials with standardized POD ascertainment remain necessary [47].

When cognitive and physical prehabilitation are coupled with targeted nutritional support (to resolve baseline deficiencies and supply substrates needed to combat inflammaging), the clinical results are synergistic [8,38]. Limited preliminary evidence suggests that selected multimodal programs may influence delirium-related outcomes, but available trials are heterogeneous and have rarely designated POD as the primary endpoint. Cognitive prehabilitation is more directly aligned with the neurocognitive vulnerability underlying POD. This approach typically involves structured exercises targeting attention, working memory, processing speed, and executive function during the period preceding surgery. The Neurobics randomized clinical trial provided an important proof of concept by evaluating preoperative cognitive exercise in older adults undergoing major non-cardiac surgery and suggested that such training may reduce the incidence of postoperative delirium [48].

Nevertheless, the evidence base remains limited, and several questions remain unresolved, including the optimal duration and intensity of training, the minimum effective exposure, adherence to unsupervised digital programs, and the identification of patients most likely to benefit. It is therefore premature to conclude that short-term cognitive training produces structural neural reorganization or directly increases cognitive reserve. A more cautious interpretation is that repeated cognitive engagement may enhance cognitive preparedness or compensatory capacity during the perioperative period, thereby increasing tolerance to transient disturbances in attention and executive function. This remains an attractive hypothesis, but one that requires confirmation in adequately powered multicenter trials with standardized delirium assessment [48]. Multimodal prehabilitation extends this concept by addressing several dimensions of vulnerability simultaneously. Contemporary programs commonly combine individualized exercise with nutritional optimization and, in some protocols, psychological or behavioral support. The PREHAB randomized clinical trial demonstrated the feasibility and clinical relevance of a four-week multimodal program before colorectal cancer surgery, adding to evidence that preoperative optimization can improve functional recovery and selected postoperative outcomes. This multidomain approach is particularly appealing in the context of delirium because frailty, malnutrition, physical deconditioning, psychological stress, and cognitive impairment frequently coexist and may exert cumulative effects on perioperative vulnerability. Delirium has rarely been specified as a primary outcome in contemporary prehabilitation trials. It therefore remains inappropriate to conclude that multimodal prehabilitation has been proven to reduce either the incidence or duration of POD. At present, its potential contribution to delirium prevention should be viewed as biologically plausible and clinically promising but still indirect. Future trials should incorporate delirium as a prespecified outcome, use validated assessment instruments over an adequate postoperative surveillance period, and determine whether improvements in physical or cognitive reserve mediate any observed reduction in delirium risk [46,48].

The time available before surgery represents an important limitation to clinical implementation. Recent intervention studies have demonstrated benefit with programs lasting approximately three to four weeks, but this interval should not be regarded as a universal or evidence-based minimum. Thus, prehabilitation should be regarded as one component of a broader perioperative strategy whose applicability is determined by the available preoperative window and the patient’s individual vulnerability, rather than as a universal prerequisite for delirium prevention [44,45,46,48].

### 3.4. Intraoperative Management and Prevention Strategies

The intraoperative period is a critical component of postoperative delirium (POD) prevention because it is the point at which modifiable anesthetic, physiological, and surgical stressors directly interact with pre-existing cerebral vulnerability. Current evidence does not support a single protective technique, drug, monitor, or hemodynamic target. Instead, intraoperative neuroprotection is best understood as an individualized strategy that limits avoidable hypnotic exposure, preserves physiological stability and oxygen delivery, provides adequate analgesia, and uses advanced cerebral monitoring selectively when the information is likely to alter management [8,33,38,49,50]. A dedicated systematic review has examined delirium-prevention strategies in vascular-surgery populations, highlighting the need for setting-specific evidence rather than uncritical extrapolation across surgical cohorts [51].

#### 3.4.1. Individualizing Anesthetic Depth and EEG Monitoring

The association between anesthetic depth, electroencephalographic (EEG) suppression, and POD is biologically plausible but clinically complex. Processed EEG indices can help identify profound cortical depression, yet burst suppression should not be interpreted as a direct cause of delirium in every patient. In older or cognitively vulnerable adults, suppression may occur at relatively low anesthetic concentrations and may therefore reflect both drug exposure and reduced cerebral reserve. The central clinical question is not whether a single numerical threshold prevents delirium but whether EEG information can help avoid disproportionate cerebral depression in an individual patient [52,53,54].

Randomized trials illustrate this uncertainty. In ENGAGES, EEG-guided anesthesia reduced anesthetic exposure only modestly and did not lower POD incidence (26.0% with guidance and 23.0% with usual care). By contrast, the BALANCED Delirium substudy reported less delirium with a lighter anesthetic target than with a deeper target (19% for BIS-50 group vs. 28% for BIS-35 group; OR, 0.58; 95% CI 0.38–0.88) [53]. More recently, ENGAGES-Canada achieved a clear reduction in volatile anesthetic exposure and cumulative EEG suppression in older adults undergoing cardiac surgery; the primary report records median MAC 0.66 versus 0.80 and suppression 4.0 vs. 11.7 min, with POD 18.15% vs. 18.10%. Together, these findings argue against a simple linear pathway in which greater anesthetic dose produces EEG suppression that directly causes delirium. The effect of depth modification is likely to depend on baseline vulnerability, surgical population, the magnitude of separation between anesthetic exposures, and competing perioperative insults [52,53,55,56].

Accordingly, processed indices such as the Bispectral Index or Patient State Index should be interpreted as adjuncts rather than autonomous therapeutic targets. Similar numerical values may represent different underlying EEG states, including organized oscillatory activity, diffuse slowing, or suppression. In high-risk patients, a more informative approach is to integrate the processed index with the frontal EEG waveform and, where available, time–frequency displays such as the density spectral array (DSA). This allows clinicians to recognize abrupt changes in cortical activity, identify sustained suppression, and relate the neurophysiological response to anesthetic dose and the broader clinical context. Attenuated frontal alpha activity may provide additional information about cerebral sensitivity, but it is influenced by age, anesthetic agent, concentration, baseline physiology, and co-administered drugs and should not be treated as a specific marker of neurodegeneration or impending delirium [52,53].

#### 3.4.2. Choice of Anesthetic Technique

Current evidence does not support a binary distinction between an intrinsically neuroprotective intravenous technique and a neurotoxic volatile technique. Although experimental studies describe agent-specific effects on oxidative stress, inflammatory signaling, mitochondrial function, and amyloid or tau-related pathways, these findings cannot be directly translated into clinical delirium risk. Both propofol and volatile anesthetics can produce excessive cortical depression, hemodynamic disturbance, and EEG suppression when administered inappropriately, whereas either technique can be delivered safely when carefully titrated. The apparent advantages of total intravenous anesthesia (TIVA) reported in some studies are difficult to separate from differences in anesthetic depth, opioid exposure, blood pressure, postoperative sedation, and delirium ascertainment. Meta-analytic comparisons of intravenous and inhalational anesthesia in older non-cardiac surgical patients have also assessed postoperative cognition; because cognitive test outcomes and delirium are not interchangeable, these findings should be treated as indirect rather than definitive POD evidence [57]. TIVA should therefore not be selected solely to prevent POD; the maintenance technique should be chosen according to the patient, procedure, and established clinical indications while avoiding unnecessary drug exposure [8,34,35,38]. A recent systematic review comparing inhalational anesthesia with target-controlled or manual TIVA focused on broader safety and recovery outcomes and does not establish a universal delirium-preventive advantage for either maintenance strategy [58].

The same principle applies to the choice between regional and general anesthesia. In older adults undergoing hip fracture surgery, the RAGA trial found no significant difference in POD between regional anesthesia without sedation and general anesthesia (6.2% vs. 5.1%), while the REGAIN trial similarly reported comparable delirium rates after spinal and general anesthesia (20.5% vs. 19.7%). These results do not diminish the value of neuraxial or peripheral regional techniques, which may improve analgesia and reduce systemic opioid requirements in selected patients. However, regional anesthesia should not be chosen solely on the expectation of intrinsic protection from delirium. Technique selection should remain individualized according to surgical requirements, contraindications, anticoagulant therapy, airway and hemodynamic considerations, patient preference, sedation requirements, and local expertise [8,33,38,59].

#### 3.4.3. Hemodynamic Stability and Cerebral Oxygenation

Preservation of systemic perfusion is a rational component of intraoperative neuroprotection, but no universal blood-pressure threshold has been validated specifically for POD prevention. Aging, chronic hypertension, cerebrovascular disease, and impaired vascular reactivity can alter cerebral autoregulation, yet the degree of vulnerability varies substantially between patients. Observational studies have associated deeper or prolonged hypotension with adverse postoperative outcomes, but delirium-specific findings remain inconsistent and are confounded by blood loss, illness severity, low cardiac output, surgical complexity, and anesthetic exposure. Therefore, an absolute mean arterial pressure below 65 mmHg, a reduction greater than 20% from baseline, or maintenance within 10% of the preoperative value should not be presented as universal delirium-prevention targets. A more defensible approach is to avoid sustained or profound hypotension and marked hemodynamic variability while identifying and treating the mechanism of instability, whether vasodilation, hypovolemia, hemorrhage, low cardiac output, arrhythmia, or excessive anesthetic exposure [8,34,35,38].

Near-infrared spectroscopy (NIRS) may extend assessment beyond systemic blood pressure by providing a trend estimate of regional frontal cerebral oxygen saturation. However, the signal does not directly measure cerebral blood flow, neuronal metabolism, or tissue ischemia and is influenced by arterial oxygenation, hemoglobin concentration, carbon dioxide tension, blood pressure, cardiac output, extracranial tissue, sensor position, and device-specific algorithms. Relative decreases of approximately 20% from baseline and absolute values near 50–55% are commonly used as intervention triggers, but these thresholds are protocol-derived rather than validated boundaries for delirium or cerebral injury. When a desaturation occurs, a structured response should verify the signal and assess head position, oxygenation, ventilation and PaCO2, perfusion pressure, hemoglobin concentration, and cardiac output. Because evidence has not shown that routine correction of NIRS values independently prevents POD, cerebral oximetry is best regarded as a selective adjunct in procedures or patients in whom the information is likely to guide management [8,35,38].

#### 3.4.4. An Integrated Approach to Intraoperative Neuroprotection

The accumulated evidence shifts the focus away from searching for a single anesthetic, monitor, or physiological target that prevents delirium. POD is more plausibly understood as the clinical consequence of cumulative perioperative stress exceeding the compensatory capacity of a vulnerable brain. Intraoperative prevention should therefore combine an appropriate anesthetic technique with avoidance of unnecessary hypnotic exposure, adequate analgesia, prompt correction of relevant physiological disturbances, and preservation of oxygen delivery. EEG and cerebral oximetry may add value when used selectively and interpreted within the wider clinical context rather than as stand-alone targets. This individualized strategy should be integrated with preoperative risk recognition and structured postoperative prevention, with the overarching aim of reducing the total burden of modifiable cerebral stress while maintaining safe and effective conditions for surgery [8,34,35,38,52,53].

### 3.5. Prevention and Treatment of Postoperative Delirium: Multicomponent Care, Selective Pharmacotherapy, and the Implementation Gap

Contemporary management of postoperative delirium (POD) is increasingly organized around prevention rather than treatment after the syndrome has become established. This approach reflects the multifactorial nature of delirium, in which acute perioperative stressors interact with pre-existing cognitive, physiological, and functional vulnerability. Once delirium develops, no single pharmacological intervention reliably reverses the underlying process or shortens its clinical course. Accordingly, current perioperative strategies prioritize early identification of susceptible patients, reduction of modifiable precipitating factors, and coordinated delivery of non-pharmacological interventions across the surgical pathway [34,35,38,60]. The objective is not to target a single hypothetical mechanism but to reduce the cumulative burden of factors capable of destabilizing cerebral homeostasis, including pain, excessive sedation, sleep disruption, immobility, dehydration, sensory deprivation, metabolic disturbance, and exposure to deliriogenic medications. When delirium nevertheless occurs, the same principles remain relevant: reversible causes should be actively sought and corrected, the environment should be adapted to support orientation and sleep–wake regulation, and pharmacological treatment should be reserved for carefully selected clinical circumstances rather than used as routine therapy [34,35,38,60].

#### 3.5.1. Multicomponent Non-Pharmacological Prevention and the Role of HELP

Among available preventive approaches, multicomponent non-pharmacological interventions have the most consistent evidence base. Their rationale is inherently compatible with the pathophysiology of delirium: because no single precipitating factor accounts for most cases, simultaneous modification of several risk domains is more plausible than isolated intervention [50]. The Hospital Elder Life Program (HELP) remains the prototypical model for this approach. Originally developed for older medical inpatients, its principles have subsequently informed delirium-prevention pathways across medical, surgical, and perioperative settings [61]. Contemporary evidence supports the underlying multicomponent strategy, although the magnitude of benefit varies according to population, intervention composition, baseline delirium risk, and fidelity of implementation [8,38,60,62]. The principal value of HELP therefore lies less in adherence to a fixed historical protocol than in its demonstration that delirium prevention requires coordinated attention to multiple, individually modest but cumulatively important determinants of cerebral vulnerability. Modern adaptations of this approach typically incorporate repeated orientation and cognitive engagement, protection of the sleep–wake cycle, early mobilization, correction of sensory impairment, adequate hydration and nutrition, medication review, pain management, and prevention of avoidable complications such as constipation and urinary retention [8,38,60,62]. Orientation should extend beyond periodically reminding patients of the date and location. Effective cognitive support involves clear communication, preservation of familiar routines where possible, access to clocks and calendars, minimization of unnecessary room transfers, and involvement of relatives or caregivers who can provide knowledge of the patient’s baseline cognitive and behavioral state. Similarly, sleep protection should be understood as an organizational intervention rather than a collection of isolated comfort measures. Reducing unnecessary nocturnal observations, avoiding overnight procedures when clinically feasible, limiting environmental noise and light, promoting daytime mobilization, and avoiding inappropriate sedative-hypnotic prescribing may be more relevant than any single sleep-promoting intervention.

Early mobilization is another central component, but its role should not be reduced to a theoretical effect on inflammatory or neurotrophic pathways. Its immediate clinical value lies in limiting deconditioning, supporting functional recovery, improving daytime activity, and reducing the cascade of immobility-related complications that can prolong exposure to the hospital environment. Sensory optimization is similarly pragmatic. Ensuring access to glasses and hearing aids may reduce perceptual ambiguity and improve communication, while hydration and nutritional assessment address common physiological precipitants of acute cognitive deterioration. Medication review should be incorporated explicitly into the bundle, with particular attention to cumulative anticholinergic burden, benzodiazepines, unnecessary sedative-hypnotics, and other centrally acting agents that may exacerbate cognitive dysfunction [34,35,38,60]. The 2023 AGS Beers Criteria provide a practical framework for identifying potentially inappropriate anticholinergic, sedative, and other centrally acting medications in older adults [63].

Importantly, the evidence should not be interpreted as showing that the HELP label itself prevents delirium. The effectiveness of any multicomponent program depends on whether its individual elements are delivered reliably to the patients who require them. A protocol documented in a hospital guideline but inconsistently executed is unlikely to reproduce the outcomes observed in structured clinical programs. Contemporary implementation research therefore shifts attention from the content of the bundle alone to its operational fidelity: who identifies high-risk patients, who activates the intervention, how adherence is monitored, and what happens when a component is missed. This distinction is particularly important in surgical wards, where shortened admissions, frequent patient transfers, competing procedural priorities, and high nursing workloads can fragment otherwise well-designed preventive pathways [8,38,60].

#### 3.5.2. The ABCDEF Bundle: ICU Liberation Rather than a POD-Specific Protocol

The ABCDEF bundle represents a related but conceptually distinct framework. Unlike HELP, which was developed primarily around delirium prevention in older hospitalized adults, ABCDEF emerged within critical care as a broader strategy for reducing the adverse consequences of mechanical ventilation, deep sedation, immobility, and prolonged critical illness. Its components address A, pain assessment/prevention/management; B, spontaneous awakening and breathing trials; C, choice of analgesia and sedation; D, delirium assessment/prevention/management; E, early mobility and exercise; F, family engagement and empowerment. The bundle therefore extends well beyond delirium itself and is better understood as an integrated model of ICU liberation and recovery rather than as a POD-specific intervention [64].

Several components have direct relevance to acute brain dysfunction. Systematic pain assessment may reduce both untreated nociceptive stress and unnecessary opioid escalation. Coordinated spontaneous awakening and breathing trials reduce exposure to prolonged deep sedation and facilitate earlier neurological assessment. The choice and titration of sedative agents may limit unnecessary cerebral depression, while repeated delirium assessment supports earlier recognition of fluctuating cognitive dysfunction. Early mobilization and family involvement address functional, environmental, and psychological contributors that are otherwise easily neglected in highly technological critical care environments. The strength of the ABCDEF framework lies in coordinating these processes rather than relying on any single component as an independent neuroprotective intervention.

Observational studies and contemporary evidence syntheses generally associate greater bundle implementation with favorable clinical outcomes, including fewer days with acute brain dysfunction and improved recovery-related outcomes. Nevertheless, the apparent dose–response relationship between bundle compliance and outcome should be interpreted cautiously. Patients who are sufficiently stable to receive multiple bundle components may differ substantially from those in whom implementation is limited by shock, severe neurological injury, refractory hypoxemia, or other forms of critical illness. Thus, higher compliance may partly reflect lower illness severity as well as the effect of the intervention itself. Recent syntheses nevertheless support coordinated care bundles as an important organizational strategy in critical care while also emphasizing heterogeneity in bundle composition and delivery [64].

Extrapolation of ABCDEF to general surgical wards or step-down units should therefore be selective rather than automatic. Principles such as pain assessment, minimization of unnecessary sedation, early mobility, repeated delirium surveillance, and family engagement are broadly transferable, but spontaneous awakening and breathing trials are inherently ICU-specific. The more relevant objective is continuity of neurocognitive care across transitions. High-risk patients should not move from an intensive, structured delirium-prevention environment to a ward where cognitive surveillance and preventive interventions abruptly disappear. Rather than reproducing the entire ABCDEF bundle outside the ICU, institutions should preserve the clinically relevant elements across postoperative transitions and adapt them to the resources and risks of each setting [8,38,64].

#### 3.5.3. Pharmacological Prevention: No Universal Delirium-Preventive Agent

The increasing biological sophistication of delirium research has not yet produced a universally effective pharmacological preventive strategy. This is unsurprising given the heterogeneity of the syndrome. A patient whose delirium is predominantly precipitated by severe infection, another exposed to deep sedation and prolonged ventilation, and a third with pre-existing cognitive impairment who develops delirium after relatively minor physiological disturbance are unlikely to respond identically to a single pharmacological intervention. The contemporary pharmacological landscape should therefore not be described as having progressed from empirical treatment to established “precision pharmacotherapy.” At present, the evidence supports a more modest conclusion: selected agents may modify delirium risk in specific clinical contexts, but none can substitute for multicomponent prevention or be recommended indiscriminately across surgical populations. Luney et al., in a 2026 network meta-analysis including 158 randomized trials and more than 41,000 older surgical patients, identified dexmedetomidine as the pharmacological intervention with the most consistent evidence for reducing postoperative delirium, whereas benefits associated with corticosteroids, melatonin-receptor agonists, parecoxib, olanzapine, and intranasal insulin were largely confined to specific clinical settings and remain insufficient to support universal prophylaxis [8,32,34,35,38]. Importantly, the 158 trials and 41,084 participants represent the entire network of randomized trials evaluating the various pharmacological strategies included in the analysis and should not be interpreted as the evidence base for dexmedetomidine alone [32]. As with any network meta-analysis, interpretation of treatment estimates depends on the assumptions underlying both direct and indirect comparisons. Validity of the treatment comparisons depends on transitivity across sufficiently comparable populations, interventions, and trial characteristics, consistency between direct and indirect evidence, and the extent of heterogeneity within each comparison. In this context, differences in surgical population, baseline delirium risk, comparator, drug dose and timing, and delirium ascertainment may act as important effect modifiers and should be considered when interpreting the pooled estimates. These considerations are especially relevant in postoperative delirium, where studies differ substantially with respect to surgical population, baseline frailty and delirium risk, anesthetic and perioperative management, comparator intervention, dexmedetomidine dose and timing of administration, and the instruments, frequency, and duration used for delirium ascertainment. Consequently, the favorable position of dexmedetomidine within the network should be interpreted as evidence of a comparatively consistent preventive signal rather than as proof of a uniform treatment effect across all surgical populations or as justification for indiscriminate prophylactic use [32,65,66].

#### 3.5.4. Dexmedetomidine: Context-Dependent Benefit Rather than a Universal Standard

Dexmedetomidine has attracted particular interest because its pharmacological profile differs from that of conventional GABAergic sedatives. Through central α_2_-adrenergic agonism, particularly within locus coeruleus pathways, it reduces sympathetic outflow and produces a state in which patients are often more readily arousable than during deeper propofol- or benzodiazepine-based sedation. These characteristics are clinically attractive in patients requiring sedation while retaining the possibility of interaction and neurological assessment. However, describing dexmedetomidine-induced sedation as equivalent to physiological non-rapid-eye-movement sleep is too definitive. Although there are electrophysiological and neurophysiological similarities, pharmacological sedation does not reproduce normal sleep architecture in its entirety [8,32,35,38,67].

Among pharmacological interventions studied for delirium prevention, dexmedetomidine has shown one of the more consistent signals of potential benefit, particularly in selected older surgical patients and in contexts where it reduces exposure to deeper or more deliriogenic sedative regimens. Meta-analyses published in 2025 and 2026 demonstrated significantly lower POD incidence with dexmedetomidine (RR 0.67, 95% CI 0.59–0.76; OR 0.46, 95% CrI 0.36–0.57) [31,32]. Importantly, its effects vary according to timing, dose, surgical population, comparator, baseline delirium risk, and whether the drug is used as an anesthetic adjunct, postoperative sedative, or low-dose nocturnal infusion. The available evidence therefore does not justify describing dexmedetomidine as the “gold standard” pharmacological prophylaxis for POD. Current guidance supports selective rather than universal use, and the potential benefit must be weighed against clinically important bradycardia and hypotension, particularly in older patients with conduction abnormalities, limited chronotropic reserve, or hemodynamic instability [8,32,35,38].

The most defensible interpretation is that dexmedetomidine may be useful when its pharmacological characteristics align with an existing clinical indication. For example, it may be preferable to benzodiazepine-heavy sedation in selected critically ill patients or may permit lighter, more interactive sedation in circumstances where ongoing sedation is otherwise necessary. This is fundamentally different from administering dexmedetomidine to every older surgical patient solely for delirium prevention. Future studies should define which phenotypes are most likely to benefit, the optimal timing and dose, and whether any preventive effect arises from direct pharmacological properties or indirectly from reduced exposure to alternative sedative agents [8,32,34,35,38,66].

#### 3.5.5. Melatonin and Circadian-Directed Strategies: Biological Plausibility, Inconsistent Clinical Evidence

Disruption of sleep and circadian organization is a prominent feature of both the perioperative environment and established delirium. Surgery, pain, anesthetic exposure, nocturnal care interventions, artificial lighting, immobility, and critical illness can all disrupt the normal temporal organization of sleep and wakefulness. These observations provide a biologically plausible rationale for circadian-directed interventions, including melatonin and melatonin-receptor agonists. Melatonin signaling is involved not only in sleep timing but also in broader neuroendocrine and immune regulation, which has encouraged investigation of these agents as potential low-risk preventive strategies.

Clinical translation, however, remains inconsistent. Trials of exogenous melatonin and melatonin-receptor agonists have differed substantially in patient population, dose, treatment timing, comparator, and delirium ascertainment. Positive findings have not been reproduced uniformly, and contemporary perioperative guidance does not support presenting melatonin-based therapy as an established standard for POD prevention. The claim that these agents are particularly effective in orthopedic or vascular surgery is also stronger than the available evidence permits. Similarly, improved sleep and prevention of delirium should not be treated as interchangeable outcomes: an intervention may influence subjective or objective sleep without necessarily preventing the multifactorial syndrome of delirium [8,32,35,38].

The more appropriate position is that circadian-directed pharmacology remains a promising but incompletely validated adjunct. Its favorable respiratory profile compared with conventional sedative-hypnotics makes further study attractive, particularly in high-risk older adults, but absence of respiratory depression does not imply absence of adverse effects or clinically relevant drug interactions. At present, preservation of circadian organization is better supported as part of a broader non-pharmacological strategy—daytime activity and light exposure, avoidance of unnecessary nocturnal disruption, minimization of sedative-hypnotic exposure, and maintenance of a recognizable day–night structure—than as a justification for routine melatonin or ramelteon administration [32,35,38,60].

#### 3.5.6. Cerebral Metabolic Vulnerability and Intranasal Insulin: A Translational Therapeutic Target

Growing evidence suggests that cerebral metabolism may contribute to POD, as insulin signaling within the central nervous system extends beyond peripheral glucose regulation. Insulin receptors are widely distributed in brain regions responsible for memory, attention, executive function, and behavior, where they regulate synaptic plasticity, neuronal energy utilization, neurotransmitter homeostasis, and cell survival. During major surgery, neuroendocrine activation, transient insulin resistance, systemic inflammation, and altered substrate utilization may interact with pre-existing cognitive impairment, vascular disease, and reduced metabolic reserve, thereby lowering the threshold for acute cerebral dysfunction [8,34,35,38,68].

Metabolic vulnerability may be particularly relevant in patients with diabetes, in whom chronic hyperglycemia, glycemic variability, insulin resistance, vascular dysfunction, oxidative stress, and persistent low-grade inflammation may converge to reduce cerebral resilience to perioperative stress. Alwesabi et al. highlighted these interacting mechanisms as potential contributors to both postoperative delirium and longer-term postoperative cognitive impairment while also emphasizing the importance of baseline metabolic and vascular comorbidity in determining individual susceptibility. Importantly, much of the available evidence remains observational, and associations between diabetes-related metabolic disturbances and POD do not establish that correction of peripheral glycemic abnormalities restores cerebral insulin signaling or directly prevents delirium. Diabetes should therefore be regarded as a clinically relevant metabolic vulnerability state rather than as evidence supporting any specific cerebral metabolic intervention [69].

Systemic insulin resistance should not be considered equivalent to impaired cerebral insulin signaling. Peripheral hyperglycemia neither confirms central insulin resistance nor indicates restoration of neuronal insulin function after glycemic correction. Experimental studies have linked impaired brain insulin signaling with disrupted phosphoinositide 3-kinase/protein kinase B (PI3K/Akt) signaling, glycogen synthase kinase-3β (GSK-3β) activation, abnormal tau phosphorylation, and impaired synaptic plasticity. Although these mechanisms overlap with pathways implicated in neurodegenerative disease, they should not be interpreted as evidence that POD represents an acute form of Alzheimer-type pathology. Rather, delirium remains a heterogeneous syndrome arising from the interaction of inflammatory, vascular, pharmacological, environmental, and metabolic stressors [8,34,38,68].

Intranasal insulin has emerged as a promising experimental strategy because it may enhance central insulin signaling while minimizing systemic insulin exposure and the risk of hypoglycemia. Most clinical experience, however, derives from studies of chronic cognitive impairment rather than perioperative care. Although these investigations suggest that central insulin responsiveness can be pharmacologically modulated, therapeutic effects have been inconsistent and appear influenced by metabolic phenotype, neurodegenerative burden, dose, treatment duration, delivery device, sex, and apolipoprotein E genotype [68]. Consequently, contemporary perioperative guidelines do not recommend intranasal insulin for delirium prevention, and current evidence is insufficient to define its optimal dose, timing, or target population [8,32,35,38,68].

Intranasal insulin therefore remains a hypothesis-generating perioperative intervention; future proof-of-concept trials should prioritize validated delirium assessment, mechanistic biomarkers, and careful differentiation of POD from longer-term postoperative neurocognitive disorders before routine clinical implementation can be considered [35,68].

#### 3.5.7. Antipsychotics: Behavioral Control Rather than Disease Modification

The role of antipsychotic medication has narrowed substantially as evidence has failed to demonstrate a meaningful effect on the underlying course of delirium. Dopamine-receptor antagonism may reduce selected behavioral manifestations, particularly severe agitation or psychotic symptoms, but it does not correct the multiple physiological processes responsible for delirium. Antipsychotics should therefore not be used for routine prophylaxis, nor should improvement in agitation be interpreted as resolution of the delirium itself [8,38,70].

The AID-ICU trial provided important contemporary evidence in this regard. In critically ill adults with established delirium, haloperidol did not improve the primary outcome compared with placebo. The study was conducted in an ICU population and was not specific to postoperative delirium, so its findings should not be extrapolated uncritically to every surgical setting. Nevertheless, it reinforces the broader conclusion that routine antipsychotic administration does not modify the natural history of delirium in a clinically meaningful way [70].

A “rescue-only” framework captures the direction of modern practice but should not be expressed in absolute terms. Antipsychotic medication may still be considered for short-term control of severe agitation, psychotic distress, or behavior that creates an immediate risk to the patient or others, particularly when the behavior prevents essential medical treatment. Before administration, clinicians should actively evaluate and address reversible contributors such as pain, hypoxemia, urinary retention, constipation, withdrawal, infection, and excessive environmental stimulation. Non-pharmacological de-escalation and the presence of familiar caregivers should be used when feasible. When medication is considered necessary, the lowest effective dose should be used for the shortest possible duration, with repeated reassessment of the indication and attention to QT prolongation, extrapyramidal effects, orthostatic hypotension, oversedation, and conditions associated with increased sensitivity to dopamine blockade [8,32,38,70]. Within this framework, treatment selection should account for the safety profile of agents used to control delirium-related agitation. In a small randomized study of 90 patients with established ICU delirium, Haspolat et al. examined QT and QTc changes during haloperidol or dexmedetomidine treatment. These findings concern an electrophysiological surrogate in a specific ICU population and do not establish comparative arrhythmia safety, superiority for delirium treatment, or applicability to routine prophylaxis of postoperative delirium [71].

Taken together, the interventions reviewed above differ substantially in their readiness for clinical implementation. Table 3 summarizes their target populations, principal supporting evidence, major risks and limitations, and current clinical status, distinguishing strategies appropriate for routine practice from those applicable only in selected clinical contexts and those that remain investigational. This distinction is clinically important because biological plausibility, or even a statistically significant effect in an individual trial, does not necessarily indicate sufficient evidence for routine perioperative implementation.

#### 3.5.8. The Implementation Gap: From Evidence-Based Bundles to Reliable Bedside Delivery

The persistence of POD despite an expanding evidence base increasingly directs attention towards implementation rather than intervention discovery alone. The problem is not simply that clinicians lack knowledge of delirium risk factors. In many institutions, the individual components of prevention are already familiar: assess cognition, avoid unnecessary sedatives, manage pain, mobilize early, correct sensory impairment, preserve sleep, and monitor for acute change. The difficulty lies in ensuring that these measures are delivered consistently across different professionals, clinical environments, and phases of care.

Delirium detection illustrates this problem. Large-scale routine-practice data demonstrate that structured tools such as the 4AT can be integrated into clinical care and that positive scores identify patients with substantially worse outcomes [20]. However, contemporary implementation studies also show considerable variation in completion rates and in the proportion of patients identified as positive when screening tools are deployed at scale [21]. Screening alone therefore cannot be considered a preventive intervention. A completed score has limited clinical value unless it initiates a defined response: confirmation of acute change, assessment for reversible causes, medication review, activation of preventive or therapeutic measures, communication during handover, and subsequent reassessment.

The same principle applies to prevention bundles. Institutional success depends on clearly defined responsibility, rapid identification of eligible patients, integration into existing workflows, monitoring of adherence, and continuity during transfers between the operating theatre, recovery unit, ICU, and surgical ward. In many settings, the most vulnerable moment may occur at the transition between teams, when information about baseline cognition, intraoperative instability, sedative exposure, or early signs of delirium is lost. High-quality neurocognitive handover should therefore be considered part of the prevention pathway.

Implementation also requires realistic adaptation to resource constraints. A program dependent on specialist geriatric teams or dedicated volunteers may not be reproducible in every hospital. The appropriate response is not to abandon multicomponent prevention, but to identify the highest-value elements that can be delivered reliably within the local system. Process measures may be particularly useful: proportion of eligible patients screened, percentage receiving sensory aids, time to mobilization, exposure to potentially deliriogenic medication, use of physical restraints, completion of medication review, and response time after a positive delirium screen. Such measures provide a more actionable assessment of program function than delirium incidence alone, which is strongly influenced by case mix and the sensitivity of detection.

**Figure 2:**
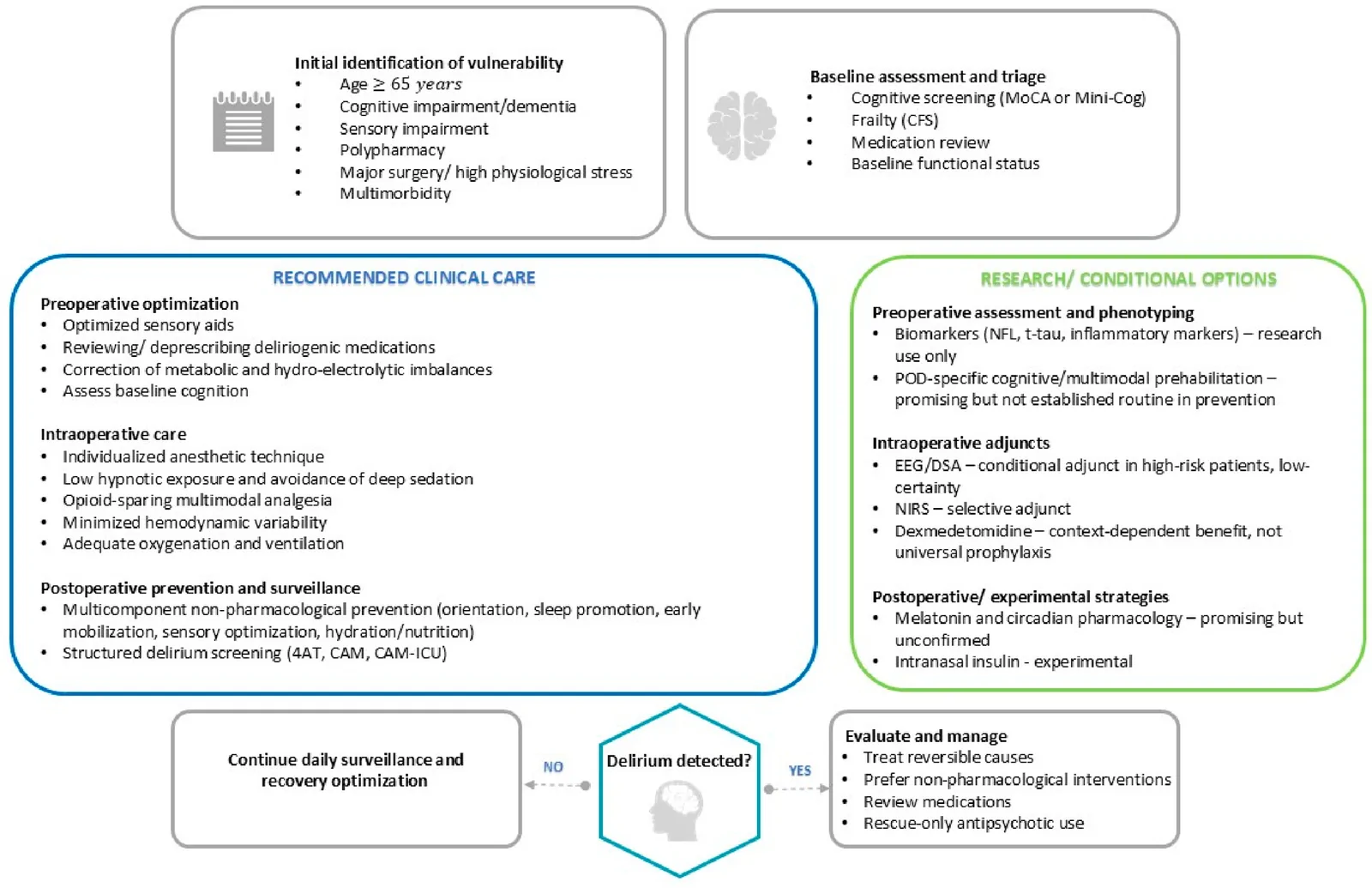
Proposed evidence-informed clinical pathway for perioperative delirium risk stratification, prevention, detection, and response. CFS: Clinical Frailty Scale; EEG: Electroencephalography; DSA: Density Spectral Array; NIRS: Near-Infrared Spectroscopy; CAM: Confusion Assessment Method; 4AT: 4 A’s Test.

Ultimately, the contemporary management of POD is characterized less by the emergence of a single transformative therapy than by a change in clinical architecture. Multicomponent non-pharmacological prevention remains the foundation of care; ICU bundles offer a framework for coordinated liberation from deep sedation and immobility; dexmedetomidine may benefit selected patients but is not universal prophylaxis; melatonin-based strategies remain promising but unconfirmed; intranasal insulin remains experimental; and antipsychotics have a restricted role in short-term behavioral control rather than disease modification. The major translational challenge is to convert these distinctions into reliable everyday practice. Future progress will depend not only on identifying new biological targets, but on designing perioperative systems capable of recognizing vulnerability, delivering preventive measures consistently, detecting delirium early, and linking every positive assessment to a timely clinical response [20,21,34,35,38,60,64,70]. A proposed evidence-informed clinical pathway detailing this proactive risk stratification and subsequent triage is outlined in Figure 2.

## 4. Limitations

This structured narrative review has several limitations inherent to its design and to the underlying postoperative delirium literature. Although a structured literature search and predefined eligibility criteria were used to identify clinically relevant evidence across the perioperative pathway, the review was not intended to provide the exhaustive coverage of a formal systematic review. Restriction to English-language publications may also have introduced language and publication-selection bias. Interpretation across studies is further complicated by heterogeneity in surgical populations, baseline vulnerability, delirium definitions and assessment instruments, surveillance periods, intervention protocols, and perioperative settings. The clinical-maturity categories presented in this review should be interpreted as an evidence-informed framework for clinical applicability rather than as formal certainty-of-evidence ratings; no formal GRADE assessment or new quantitative meta-analysis was performed. Particular caution is warranted when interpreting context-dependent or emerging strategies, including pharmacological prophylaxis, processed electroencephalographic monitoring, cerebral oximetry, and biomarker-guided approaches, because their apparent effects may vary according to population, comparator, intervention protocol, and delirium ascertainment. These considerations support cautious, context-specific interpretation of the available evidence while highlighting areas in which further prospective validation is needed.

## 5. Conclusions and Future Directions in Perioperative Neuroprotection

Postoperative delirium should be understood as a multifactorial manifestation of perioperative stress exceeding the adaptive capacity of a vulnerable brain, rather than as an isolated consequence of anesthesia or advanced age. This framework explains the heterogeneity of the evidence and the limited effectiveness of interventions targeting a single mechanism. Effective neuroprotection therefore requires coordinated care across the perioperative pathway. Preoperative frailty and cognitive assessment can identify vulnerable patients and guide optimization of modifiable risks. Intraoperatively, no anesthetic technique, processed-EEG target, blood-pressure threshold, or cerebral oximetry strategy has demonstrated universal superiority; management should instead minimize unnecessary hypnotic exposure, provide adequate analgesia, and preserve physiological stability and oxygen delivery. Postoperatively, multicomponent non-pharmacological prevention remains the most consistently supported approach, while pharmacological interventions should be used selectively. Dexmedetomidine may benefit specific populations, whereas melatonin-based strategies and other emerging interventions require further validation. Antipsychotics should be reserved for short-term symptom control when severe distress or immediate safety risk persists after reversible causes and non-pharmacological measures have been addressed. The principal challenge is now the reliable implementation of evidence-based care across clinical transitions. Delirium screening is meaningful only when linked to timely evaluation, management, communication, and reassessment.

By organizing perioperative strategies according to clinical maturity and linking them to an evidence-informed clinical pathway, this review provides a practical framework for distinguishing interventions suitable for routine care from those appropriate only in selected contexts or requiring further validation. Future research should prioritize standardized outcome measurement, biologically informed phenotyping, validated multimodal prediction models, and adequately powered pragmatic trials evaluating implementation fidelity, cost-effectiveness, and long-term cognitive and functional outcomes.

## Figures and Tables

**Table 1:** Eligibility criteria for the structured narrative review.

Inclusion Criteria	Exclusion Criteria
Study Type: RCTs; prospective/retrospective observational studies; systematic reviews/meta-analyses; narrative reviews; guidelines/consensus statements; selected clinically or mechanistically relevant studies	Case reports; editorials/letters without substantive evidence; conference abstracts without peer-reviewed full text
Population: Adults ≥18 years undergoing elective or emergency surgery; broader adult clinical populations permitted when directly relevant to POD mechanisms, treatment, safety, or implementation	Exclusively pediatric populations/emergence delirium
Outcome/evidence: POD as a primary, secondary, or substantial outcome/topic. Perioperative neurocognitive disorders, mechanistic studies, biomarkers, prehabilitation, anesthesia/physiology, ICU delirium treatment or safety, and implementation evidence when directly informative for POD interpretation	No substantive relevance to delirium, perioperative neurocognition, or the defined review domains. Evidence with only remote or incidental relevance to the review question
Recency: Articles published between 1 January 2021 and 26 September 2026 *	Records outside the contemporary window, except separately identified foundational sources
Language: English	Non-English records

* The 2021–2026 window applies to the publication date of records retrieved in the primary database search. Reviews published in this interval could include earlier primary studies. Selected pre-2021 primary publications were cited separately for foundational definitions, instruments, landmark trials, or enduring concepts; overlapping evidence was interpreted qualitatively without counting review-level and study-level reports as independent findings.

**Table 2:** Risk Factors for Postoperative Delirium [34].

Category	Risk Factor	Modifiable
Predisposing (Patient)	Advanced chronological age (≥65 years)	Non-modifiable
	Pre-existing cognitive impairment/Dementia	Non-modifiable
	Physical frailty	Partially modifiable (prehabilitation may improve general functional reserve)
	Baseline sensory impairment (visual/auditory)	Modifiable (via corrective devices)
	Polypharmacy and high anticholinergic burden	Modifiable (via proactive deprescribing)
Precipitating (System)	Major surgical trauma (vascular, orthopedic)	Non-modifiable
	Intraoperative hypotension/ Cerebral hypoperfusion	Potentially addressable (avoid sustained/profound hypotension and marked variability, treat the cause)
	Deep anesthetic planes/EEG burst suppression	Potentially addressable (conditional monitoring adjunct)
	Untreated nociceptive stress (Pain)	Modifiable (via opioid-sparing multimodal analgesia)
	ICU sleep fragmentation and prolonged immobility	Potentially addressable (via ABCDEF/HELP bundles)

**Table 3:** Clinical maturity of perioperative strategies for postoperative delirium prevention and management.

Clinical Maturity	Intervention/Strategy	Target Population/Context	Main Supporting Evidence	Overall Evidence Support *	Main Risks/Limitations	Current Clinical Status
Recommended for routine practice	Multicomponent non-pharmacological prevention (HELP-based principles)	Older and/or high-risk surgical patients across perioperative settings	Consistent support from guideline-based care, systematic reviews, and multicomponent intervention studies; benefit depends on implementation fidelity [8,38,60,62].	Moderate-robust	Resource intensity; variable adherence; effect depends on coordinated delivery of multiple components	Core preventive strategy; should form the foundation of POD prevention
	Structured delirium surveillance linked to a clinical response pathway	Older surgical patients and other high-risk perioperative populations; ICU and ward settings	Validated tools such as 4AT/CAM/CAM-ICU improve recognition; implementation data show that screening is most useful when positive results trigger reassessment and management [20,21].	Robust for detection; indirect for prevention	Incomplete screening, inconsistent follow-up, and false reassurance when screening is treated as an isolated task	Recommended as part of structured perioperative care, not as a stand-alone preventive intervention
	Medication review and reduction of deliriogenic exposure	Older, frail, cognitively vulnerable, and polypharmacy patients	Guidelines and observational evidence support minimizing anticholinergic burden, unnecessary benzodiazepines, sedative-hypnotics, and other centrally acting drugs [8,29,38,63].	Moderate	Complete avoidance may not be feasible; effects vary by indication, dose, and cumulative exposure	Routine component of risk reduction and multicomponent prevention
	Early mobilization, sensory optimization, hydration/nutrition, sleep protection, and adequate analgesia	Most patients at increased POD risk	Supported principally as components of multicomponent prevention bundles and guideline-directed care rather than as isolated interventions [8,38,60,62].	Moderate	Individual component effects are difficult to isolate; delivery is sensitive to staffing and workflow	Routine bundle components
Selected contexts/populations	ABCDEF bundle/ICU liberation principles	Critically ill postoperative patients, especially those requiring mechanical ventilation or ongoing sedation	Observational studies and contemporary syntheses associate greater bundle implementation with fewer days of acute brain dysfunction and improved recovery-related outcomes [64].	Moderate	Not all components are transferable outside ICU; confounding by illness severity and variable bundle fidelity	Appropriate in ICU settings; transferable elements should be adapted across transitions of care
	Processed EEG-guided anesthetic titration	Older or cognitively vulnerable patients when depth information is likely to alter anesthetic management	Randomized evidence is mixed: ENGAGES and ENGAGES-Canada showed no reduction in POD, whereas BALANCED Delirium suggested benefit from lighter anesthetic exposure [52,53,54,55,56].	Moderate	Device/index heterogeneity; processed values are not equivalent to underlying EEG states; no universal protective threshold	Selective monitoring adjunct; not an autonomous delirium-prevention target
	Cerebral oximetry (NIRS)	Selected high-risk patients or procedures where cerebral oxygenation trends may guide management	Physiologically plausible and useful for trend monitoring, but evidence does not show that routine correction of NIRS values independently prevents POD [8,35,38].	Limited	Extracranial contamination, device variability, multiple physiologic determinants, and unvalidated delirium-specific thresholds	Selective adjunct; not recommended as a universal POD-prevention strategy
	Dexmedetomidine	Selected older surgical patients and critically ill patients in whom sedation is otherwise indicated	Multiple meta-analyses, including a 2026 network meta-analysis, show one of the most consistent pharmacological signals for POD reduction [31,32].	Moderate	Bradycardia, hypotension, protocol heterogeneity, and uncertain optimal dose/timing	May be considered selectively; not universal prophylaxis
	Melatonin/melatonin-receptor agonists	Selected older patients with prominent sleep–wake disruption or high delirium risk	Biologically plausible with some positive trials and meta-analytic signals, but findings remain inconsistent across populations, agents, doses, and assessment methods [8,32,35,38].	Limited	Heterogeneous regimens; inconsistent replication; improvement in sleep does not necessarily translate into delirium prevention	Promising adjunct; insufficient evidence for routine prophylaxis
	Antipsychotics (e.g., haloperidol)	Established delirium with severe agitation, psychotic distress, or immediate safety risk after reversible causes have been addressed	AID-ICU did not demonstrate disease-modifying benefit; contemporary guidance restricts use to short-term symptom control [8,38,70]. A small ICU study provides limited comparative QT/QTc surrogate data; it does not establish comparative arrhythmia safety [71].	Moderate evidence against routine prophylaxis or disease modification	QTc prolongation, extrapyramidal effects, orthostatic hypotension, oversedation; increased sensitivity in selected neurologic disorders	May be considered short-term for immediate safety risk after reversible causes have been addressed; not recommended for routine prophylaxis or disease modification
Experimental/requires validation	Prehabilitation, particularly cognitive or multimodal programs for POD prevention	Older or frail patients undergoing elective surgery with sufficient preoperative time	Physical and multimodal prehabilitation improves functional reserve; Neurobics provides proof-of-concept for cognitive training, but POD is rarely a primary endpoint and evidence remains limited [44,45,46,48].	Preliminary	Heterogeneous interventions, adherence challenges, uncertain minimum duration, limited applicability in urgent surgery	Promising but not established specifically for POD prevention
	Blood-based biomarker-guided risk stratification (e.g., NfL, tau, inflammatory markers)	Research cohorts and high-risk surgical populations undergoing phenotyping	Observational studies associate biomarkers with vulnerability and perioperative neural injury, but no validated diagnostic thresholds or treatment-response algorithms exist [34,35,42,43].	Preliminary	Assay variability, timing dependence, influence of age/renal function/comorbidity, uncertain incremental value and cost-effectiveness	Research use; not ready for routine clinical decision-making
	Intranasal insulin/cerebral metabolic targeting	Investigational use in older or cognitively vulnerable surgical patients	Recent network meta-analysis suggests a potentially large effect signal, but perioperative evidence is sparse and derives from limited clinical data with uncertain generalizability [8,32,35,38,68].	Preliminary	Uncertain dose, timing, target population, delivery methods, and external validity	Experimental; requires prospective validation before clinical implementation

* Evidence-support descriptors represent a qualitative appraisal based on study design, consistency, directness, replication, safety, clinical applicability, feasibility, and guideline support; they are not formal GRADE ratings. POD: postoperative delirium; HELP: Hospital Elder Life Program; CAM: Confusion Assessment Method; CAM-ICU: Confusion Assessment Method for the ICU; EEG: electroencephalography; NIRS: near-infrared spectroscopy; NfL: neurofilament light chain; ICU: intensive care unit.

## Data Availability

The original contributions presented in the study are included in the article, further inquiries can be directed to the corresponding author.

## Supplementary Materials

The following supporting information can be downloaded at: https://www.mdpi.com/article/10.3390/diagnostics16213518/s1, Figure S1: PRISMA-style flow diagram illustrating identification and selection of literature for the structured narrative review; Table S1: Complete database-specific search strategies; Table S2: Standardized evidence extraction and evidence map.
