# Supplementary material for: Postoperative Delirium Across the Perioperative Pathway: A Structured Narrative Review of Clinical Maturity and Implementation Priorities
Source: Diagnostics (Basel). 2026 Oct 27;16(21):3518. doi: 10.3390/diagnostics16213518 (PMC13762076; doi:10.3390/diagnostics16213518)
Supplement: Supplementary file 1 [file diagnostics-16-03518-s001.zip › Supplementary Table S1.pdf]

Supplementary Table S1. Complete database-specific search strategies

Postoperative Delirium Across the Perioperative Pathway: A Structured Narrative Review of Clinical Maturity and Implementation Priorities

Database/platform	Execution date	Coverage	Search strategy	Filters	Records retrieved	Export
PubMed/MEDLINE	26.09.2026	01.01.2021–26.09.2026	PubMed Advanced Search:<br>("Delirium"[Mesh]<br>OR delirium[tiab]<br>OR delirious[tiab]<br>OR "acute confusional state"[tiab]<br>OR "acute confusional states"[tiab])<br>AND<br>("Perioperative Care"[Mesh]<br>OR "Perioperative Period"[Mesh]<br>OR "Postoperative Period"[Mesh]<br>OR "Surgical Procedures, Operative"[Mesh]<br>OR "Anesthesia"[Mesh]<br>OR postoperat*[tiab]<br>OR perioperat*[tiab]<br>OR postsurg*[tiab]<br>OR postanesth*[tiab]<br>OR postanaesth*[tiab]<br>OR surgery[tiab]<br>OR surgical[tiab]<br>OR anesthesia[tiab]<br>OR anaesthesia[tiab])<br>AND<br>("2021/01/01"[Date - Publication] :<br>"2026/09/26"[Date - Publication])	No electronic filters;<br>English at screening	5964	NBIB
Web of Science Core Collection	26.09.2026	01.2021–09.2026	Web of Science Core Collection Advanced Search:<br>TS=((deliri*<br>OR "acute confusional state*"	Publication period: January 2021- September 2026;<br>English at screening	7511	RIS

			OR "acute confusion"<br>OR "emergence delirium")<br>AND<br>(postoperativ*<br>OR perioperativ*<br>OR postsurg*<br>OR "post-surg*"<br>OR postanesthe*<br>OR postanaesthe*<br>OR "post-anesthe*"<br>OR "post-anaesthe*"<br>OR surg*<br>OR operativ*<br>OR anesthe*<br>OR anaesthe*))			
Cochrane Central Register of Controlled Trials (CENTRAL)	26.09.2026	2021–2026	Cochrane Library Search Manager:<br>#1 MeSH descriptor: [Delirium] explode all trees<br>#2 (delirium OR delirious OR "acute confusional state" OR "acute confusional states"):ti,ab,kw<br>#3 #1 OR #2<br>#4 MeSH descriptor: [Perioperative Care] explode all trees<br>#5 MeSH descriptor: [Perioperative Period] explode all trees<br>#6 MeSH descriptor: [Postoperative Period] explode all trees<br>#7 MeSH descriptor: [Surgical Procedures, Operative] explode all trees	No electronic filters;<br>English at screening	3675	RIS

			#8 MeSH descriptor: [Anesthesia] explode all trees<br><br>#9 (postoperat* OR perioperat* OR postsurg*<br>OR postanesth* OR postanaesth*<br>OR surgery OR surgical OR anesthesia OR anaesthesia):ti,ab,kw<br><br>#10 #4 OR #5 OR #6 OR #7 OR #8 OR #9<br><br>#11 #3 AND #10			
Scopus	26.09.2026	2021–2026	Advanced Title/Abstract/Keyword search:<br><br>TITLE-ABS-KEY(delirium<br>OR delirious<br>OR "acute confusional state"<br>OR "acute confusional states")<br>AND<br>TITLE-ABS-KEY(postoperat*<br>OR perioperat*<br>OR postsurg*<br>OR postanesth*<br>OR postanaesth*<br>OR surgery<br>OR surgical<br>OR anesthesia<br>OR anaesthesia)<br>AND PUBYEAR AFT 2020<br>AND PUBYEAR BEF 2027	No electronic filters;<br><br>English at screening	9198	RIS
