# Supplementary material for: Postoperative Delirium Across the Perioperative Pathway: A Structured Narrative Review of Clinical Maturity and Implementation Priorities
Source: Diagnostics (Basel). 2026 Oct 27;16(21):3518. doi: 10.3390/diagnostics16213518 (PMC13762076; doi:10.3390/diagnostics16213518)
Supplement: Supplementary file 1 [file diagnostics-16-03518-s001.zip › Supplementary Table S2.pdf]

Supplementary Table S2. Standardized evidence extraction and evidence map

Postoperative Delirium Across the Perioperative Pathway: A Structured Narrative Review of Clinical Maturity and Implementation Priorities

Purpose: This evidence map was created during manuscript revision to verify the revised narrative synthesis, quantitative statements, and the clinical-maturity classifications in Table 3. Table 3 categories remain a pragmatic author interpretation, not formal GRADE ratings

Scope: S2A–S2B prioritize primary studies, systematic reviews/meta-analyses, guidelines, consensus statements, and narrative syntheses that directly support quantitative claims, major perioperative interpretations, or Table 3 classifications.

"NR" = not reported/retrieved in the source methods used for this extraction; "NA" = not applicable.

S2A. Primary studies and implementation evidence

ID	Ref.	Clinical domain	Design	Setting/population	N	Intervention/exposure	Comparator	Delirium instrument	Assessment frequency/period	Effect measure / principal result	Harms/safety	Key limitations/directness
P01	[20] A. Anand, M. Cheng, T. Ibitoye, A. M. J. Maclullich, and E. R. L. C. Vardy (2022) DOI: 10.1093/ageing/afac051	Delirium ascertainment/implementation	Two-centre retrospective routine-data cohort	General acute hospitals; emergency admissions age ≥65 y (Lothian and Salford, UK)	82,770 emergency admissions	Routine 4AT at hospital admission; exposure = 4AT category	4AT 0 vs 1–3 vs ≥4; between-site implementation	4AT	Admission assessment as routine care; ~3-y data period	Completion 64% overall (77% vs 49% by site); 4AT ≥4: 18% vs 25%. 30-d mortality aOR vs 4AT 0: 5.53 (95% CI 4.99–6.13) and 3.39 (2.98–3.87).	NA (observational implementation study)	Nonrandomized; emergency/general hospital population; screening positivity is not equivalent to adjudicated POD; not a prevention study.
P02	[29] H. L. Moellmann, S. Boulghoudan, J. Kuhlmann, L. Rahm, and H. Frohnhofen (2025) DOI: 10.1007/s00391-024-02388-z	Medication burden/risk stratification	Prospective clinical cohort	University Hospital Düsseldorf; adults ≥70 y across 4 surgical disciplines	421 enrolled; ACB data available for 413	Preoperative anticholinergic burden (ACB score ≥3)	ACB <3	4AT, Nu-DESC, CAM/CAM-ICU in the underlying prospective cohort	Postoperative delirium assessed daily	POD 25.6% vs 11.0%; OR 2.79 (95% CI 1.31–5.97) for ACB ≥3.	NA (exposure study)	Observational association; residual confounding; single-centre cohort; analytical denominators differ from total enrollment; does not prove benefit from deprescribing.
P03	[44] A. E. M. Berkel et al. (2022) DOI: 10.1097/SLA.0000000000004702	Prehabilitation	Randomized clinical trial	Community-based prehabilitation before elective colorectal resection; high-risk older patients	57 randomized (28 intervention, 29 usual care)	Personalized supervised exercise, 3 wk (9 sessions)	Usual care	No dedicated POD ascertainment reported as a principal outcome	NA for POD; complications assessed through 30 d	Any postoperative complication: 42.9% vs 72.4%; RR 0.59 (95% CI 0.37–0.96).	No major between-group safety signal reported in the main outcome summary	Small trial; outcome was postoperative complications, not POD; indirect evidence for delirium prevention.
P04	[45] J. C. Woodfield, K. Clifford, G. A. Wilson, F. Munro, and J. C. Baldi (2022) DOI: 10.1111/sms.14130	Prehabilitation	Randomized clinical trial	Major abdominal surgery; adults 45–85 y	63 randomized; 46 completed both CPETs; 50 clinical follow-up	4-wk preoperative HIIT (14 sessions)	Standard care	No POD-specific instrument (delirium not a prespecified outcome)	NA for POD	ΔVO2peak between-group difference 2.73 mL/kg/min (95% CI 1.53–3.93); no statistically significant difference in clinical outcomes.	No major trial safety signal highlighted; exercise tolerability limits applicability	Fitness trial; underpowered for clinical outcomes; no delirium endpoint; indirect evidence.
P05	[46] C. J. L. Molenaar et al. (2023) DOI: 10.1001/jamasurg.2023.0198	Prehabilitation	International multicentre randomized clinical trial (PREHAB)	Colorectal cancer surgery	251 intention-to-treat (123 intervention, 128 standard care)	4-wk multimodal prehabilitation: HIIT + nutrition + psychological support ± smoking cessation	Standard perioperative care	POD not a principal/prespecified outcome	NA for POD	Severe complications (CCI >20): 17.1% vs 29.7%; OR 0.47 (95% CI 0.26–0.87).	7/123 (5.7%) intervention-related adverse events, mainly light-headedness/nausea; no serious intervention-related events reported.	Trial stopped early during COVID-19; delirium not primary; indirect evidence for POD.

ID	Ref.	Clinical domain	Design	Setting/population	N	Intervention/exposure	Comparator	Delirium instrument	Assessment frequency/period	Effect measure / principal result	Harms/safety	Key limitations/directness
P06	[48] M. L. Humeidan et al. (2021) DOI: 10.1001/jamasurg.2020.4371	Cognitive prehabilitation	Randomized clinical trial (Neurobics)	Older adults undergoing major noncardiac surgery	268 randomized; 251 primary analysis (125 vs 126)	Preoperative home-based cognitive exercise	Usual care	Brief CAM + Memorial Delirium Assessment Scale + structured chart review	Postoperative day 0–7 or discharge	POD 14.4% vs 23.0%; unadjusted P=.08; adjusted incidence rate ratio 0.58 (95% CI 0.33–0.99).	No major intervention-related harms emphasized	Single proof-of-concept trial; variable adherence (median 4.6 h); some patients had chart review only; requires replication.
P07	[52] A. Deschamps et al. (2024) DOI: 10.1001/jama.2024.8144	Processed EEG / anesthetic depth	Multicentre randomized clinical trial (ENGAGES-Canada)	4 Canadian hospitals; adults ≥60 y undergoing cardiac surgery	1,140 randomized; 1,131 assessed for primary outcome	EEG-guided anesthetic administration	Usual anesthetic care	Validated delirium assessment protocol in trial	Postoperative days 1–5	POD 18.15% vs 18.10%; difference 0.05% (95% CI −4.57 to 4.67). Median MAC 0.66 vs 0.80; EEG suppression 4.0 vs 11.7 min.	Medical complications 11.3% vs 12.7%; no intraoperative awareness reported.	Cardiac-surgery population; reduced anesthetic exposure/suppression did not translate into lower POD.
P08	[53] L. A. Evered et al. (2021) DOI: 10.1016/j.bja.2021.07.021	Processed EEG / anesthetic depth	Randomized clinical trial; BALANCED Delirium substudy	8 centres in Australia, China/Hong Kong and USA; at-risk major surgery	655 eligible; 547 enrolled in POD substudy; 515 final analytic cohort	Lighter anesthesia target (BIS 50)	Deeper target (BIS 35)	Predominantly 3D-CAM; CAM-ICU when in ICU	Daily on first 5 postoperative days	POD 19% vs 28%; OR 0.58 (95% CI 0.38–0.88).	No delirium-specific safety advantage established; parent trial addressed broader complications/mortality	Substudy/analytic exclusions; target separation and population differ from other EEG trials; not directly comparable with EEG-guidance vs usual-care designs.
P09	[54] P. L. Purdon et al. (2015) DOI: 10.1093/bja/aev213	EEG physiology	Retrospective observational EEG analysis	Adults 18–90 y receiving propofol or sevoflurane general anesthesia	155 (60 propofol, 95 sevoflurane)	Age/anesthetic-related EEG spectral characteristics	Across age and anesthetic groups	No POD outcome	Stable maintenance EEG epochs	Age-related changes in frontal EEG power/structure; not a delirium-effect estimate.	NA	Physiology study; no delirium outcome; indirect support for age-dependent interpretation of processed EEG.
P10	[55] T. G. Short et al. (2019) DOI: 10.1016/S0140-6736(19)32315-3	Anesthetic depth	International randomized controlled trial (BALANCED parent)	Older, high-risk adults undergoing major surgery	6,644 randomized	BIS target 50	BIS target 35	Delirium was not the parent trial primary outcome	Parent trial follow-up; POD substudy separately reported in P08	Primary parent outcome was 1-y mortality; supports feasibility/exposure separation rather than a standalone POD estimate.	Broader perioperative complications monitored	Indirect for POD; delirium inference should rely on dedicated substudy P08.
P11	[56] T. S. Wildes et al. (2019) DOI: 10.1001/jama.2018.22005	Processed EEG / anesthetic depth	Single-center randomized clinical trial (ENGAGES)	Barnes-Jewish Hospital; adults ≥60 y undergoing major surgery under general anesthesia	1,232 randomized; 1,213 assessed	EEG-guided anesthetic administration	Usual anesthetic care	CAM/CAM-ICU plus structured assessment protocol	Postoperative days 1–5	POD 26.0% vs 23.0%; absolute difference 3.0% (95% CI −2.0 to 8.0), P=.22.	Adverse events included movement, awareness, PONV, medical complications and death; no preventive benefit for POD.	Single center; modest exposure separation; does not support a universal EEG target.
P12	[33] T. Li et al. (2022) DOI: 10.1001/jama.2021.22647	Anesthetic technique	Multicenter randomized clinical trial (RAGA)	9 university hospitals in southeast China; adults ≥65 y with hip fracture	950 randomized; 941 evaluable	Regional anesthesia without sedation	General anesthesia	CAM	Daily during first 7 postoperative days	POD 6.2% vs 5.1%; risk difference 1.1% (95% CI −1.7 to 3.8); RR ≈1.2 (95% CI 0.7–2.0).	Adverse-event episodes similar overall; hypotension and nausea/vomiting among reported events.	Low event rate; regional setting; majority recruited at one hospital; technique-specific results not universally generalizable.
P13	[59] M. D. Neuman et al. (2021) DOI: 10.1056/NEJMoa2113514	Anesthetic technique	Pragmatic multicenter randomized clinical trial (REGAIN)	46 hospitals in USA/Canada; previously ambulatory adults ≥50 y with hip fracture	1,600 randomized	Spinal anesthesia	General anesthesia	3D-CAM	Once daily for first 3 postoperative days	POD 20.5% vs 19.7%; RR 1.04 (95% CI 0.84–1.30).	Overall adverse events similar; substantial crossover from spinal assignment to general anesthesia (~15%).	Pragmatic design/crossover; delirium secondary outcome; hip-fracture population.

ID	Ref.	Clinical domain	Design	Setting/population	N	Intervention/exposure	Comparator	Delirium instrument	Assessment frequency/period	Effect measure / principal result	Harms/safety	Key limitations/directness
P14	[61] S. K. Inouye et al. (1999) DOI: 10.1056/NEJM199903043400901	Multicomponent nonpharmacological prevention	Prospective matched controlled clinical study (foundational HELP)	General medicine service, teaching hospital; age ≥70 y	852	Protocols targeting 6 delirium risk factors (cognition, sleep, immobility, vision, hearing, dehydration)	Usual care units	CAM-based delirium ascertainment	Daily through hospitalization/discharge	Delirium 9.9% vs 15.0%; matched OR 0.60 (95% CI 0.39–0.92); adherence 87%.	No major intervention-related harms; resource/staff intensity	Nonsurgical medical population; nonrandomized matched design; foundational and indirect for modern perioperative pathways.
P15	[70] N. C. Andersen-Ranberg et al. (2022) DOI: 10.1056/NEJMoa2211868	Antipsychotic treatment	International multicenter blinded placebo-controlled RCT (AID-ICU)	16 ICUs in 5 European countries; adults with established delirium	1,000 randomized; 987 final analyses	IV haloperidol 2.5 mg TID + PRN	Placebo	CAM-ICU or ICDSC	Screened at least twice daily; treatment paused after 2 consecutive negative assessments	Days alive/out of hospital at 90 d: 35.8 vs 32.9; adjusted MD 2.9 d (95% CI −1.2 to 7.0), P=.22.	Serious adverse reactions 11 vs 9; QTc-related discontinuation 2.4% vs 1.4%.	Established ICU delirium, not POD-specific and not prophylaxis; primary endpoint was not delirium resolution.
P16	[71] A. Haspolat, F. Polat, and A. Ş. Köprülü (2024) DOI: 10.2478/jccm-2024-0027	Antipsychotic safety / ICU delirium	Single-center prospective randomized study	ICU patients with established delirium; Turkey	90 (45 haloperidol, 45 dexmedetomidine)	Haloperidol infusion	Dexmedetomidine infusion	Delirium diagnosis in ICU; specific diagnostic instrument not clearly reported in the accessible main report used for this map	QT/QTc measured before and within 24 h after drug administration	QTc: haloperidol 409.4±50.9 to 427.3±45.9 ms (P=.020); dexmedetomidine 409.4±63.1 to 409.8±49.7 ms (P=.974).	QT/QTc prolongation surrogate; ICU mortality 53.3% overall; hemodynamic changes reported.	Small, single-center, high-acuity ICU cohort; surrogate electrophysiological endpoint; does not establish comparative arrhythmia safety, delirium efficacy, or POD prophylaxis.

S2B. Reviews, meta-analyses, guidelines, consensus statements, and key narrative syntheses

ID	Ref.	Review / evidence-synthesis type	Final search date	No. studies/participants	Population coverage	Intervention/exposure/comparator	Delirium ascertainment coverage	Appraisal method	Principal finding used in manuscript	Harms/safety	Important overlap with other included reviews	Key limitations/directness
R01	[7] L. Evered et al. (2018) DOI: 10.1097/ALN.0000000000002334	Consensus nomenclature recommendations	NA (consensus nomenclature paper)	NA	Perioperative cognitive change after anesthesia/surgery	Definitions/nomenclature, not an intervention comparison	Distinguishes delirium from delayed neurocognitive recovery and postoperative NCD	Expert consensus/nomenclature working group	Defines delayed neurocognitive recovery within 30 d and postoperative NCD from 30 d to 12 mo after surgery; delirium remains a distinct acute fluctuating syndrome.	NA	Foundational terminology used across contemporary reviews/guidelines.	Not an evidence-effect synthesis.
R02	[8] C. Aldecoa et al. (2024) DOI: 10.1097/EJA.0000000000001876	Evidence- and consensus-based clinical guideline update	1 Apr 2015–28 Feb 2022	Multiple evidence reviews; exact aggregate study count not presented as one pooled set	Adults undergoing surgery; POD prevention, detection and management	Multicomponent care, anesthetic/physiologic management, drugs, monitoring	For eligible POD studies: validated tool ≥1/day for ≥3 d starting PACU/day of surgery or by POD1	Evidence review + consensus grading; update of 2017 guideline	Supports risk assessment, validated screening and multicomponent prevention; no single drug/monitor/target universally prevents POD.	Safety and feasibility incorporated into recommendations.	Direct update/supersession of R08 [38]; overlaps with R13/R14/R18/R19 and major RCTs.	Guideline synthesis; broad heterogeneity; recommendations combine evidence and expert consensus.
R03	[21] R. S. Penfold et al. (2024) DOI: 10.1111/jgs.18751	Systematic review (routine detection tools)	1980–31 Dec 2022	22 research studies + 4 audit reports; >500,000 patients	Large general acute hospital populations (n≥1,000); admission/inpatient/postoperative use	Validated routine delirium detection tools	CAM, 4AT, DOSS, bCAM, NuDESC, ICDSC; timing/frequency varied widely	Risk-of-bias assessment; all but 2 studies moderate–high risk	Completion 19–100%; positive-score rates varied markedly by tool, population and timepoint; implementation performance differs from diagnostic-accuracy studies.	NA	Includes/overlaps the same routine implementation domain as P01 [20].	Heterogeneous settings, tool deployment and denominators; detection performance is not prevention efficacy.
R04	[31] Y. Zhong, Z. Ren, J. Gao, X. He, and Q. Li (2025) DOI: 10.1186/s12871-025-03264-y	Systematic review + meta-analysis + trial sequential analysis	Inception–1 Nov 2024	32 RCTs; 6,046 participants	Cardiac surgery	Dexmedetomidine prophylaxis/adjunct	POD incidence; individual trial instruments/timing varied	PRISMA; RCT-focused risk-of-bias assessment + TSA	POD RR 0.67 (95% CI 0.59–0.76); event rates 10.8% vs 16.3%; I^2=42%.	Secondary focus included postoperative atrial fibrillation; bradycardia/hypotension remain clinically relevant across dexmedetomidine literature.	Substantial overlap with cardiac dexmedetomidine trials included in R05 [32]; partial overlap with R22 [65] depending on anesthesia context.	Cardiac-surgery-specific; trial protocols, dose/timing and comparators heterogeneous.

ID	Ref.	Review / evidence-synthesis type	Final search date	No. studies/participants	Population coverage	Intervention/exposure/comparator	Delirium ascertainment coverage	Appraisal method	Principal finding used in manuscript	Harms/safety	Important overlap with other included reviews	Key limitations/directness
R05	[32] M. Luney et al. (2026) DOI: 10.1136/bmj-2025-085539	Systematic review + Bayesian network meta-analysis	Inception–4 Mar 2024	158 RCTs; 41,084 participants; 52 drug interventions	Adults ≥60 y undergoing surgery under general or regional anesthesia	Pharmacological prevention strategies	Required validated delirium assessment tool; timing varied among trials	Duplicate screening/extraction; Cochrane RoB 2; CINeMA certainty assessment	Dexmedetomidine OR 0.46 (95% CrI 0.36–0.57); other signals included corticosteroids, melatonin receptor agonists, parecoxib, olanzapine and intranasal insulin.	Hypotension and bradycardia more common with dexmedetomidine; PONV reduced.	Umbrella network includes many trials also represented in R04 [31], R22 [65], and melatonin/insulin literatures.	Network assumptions (transitivity/consistency); diverse surgeries, regimens, comparators and ascertainment; treatment-ranking signals may not generalize uniformly.
R06	[34] C. H. Ormseth, S. C. LaHue, M. A. Oldham, S. A. Josephson, E. Whitaker, and V. C. Douglas (2023) DOI: 10.1001/jamanetworkopen.2022.49950	Systematic review of prognostic/risk-factor studies	Inception–Dec 2021	315 studies; 101,144 patients	Adult delirium across clinical settings; prospective cohort/case-control studies	Predisposing/precipitating factors	In-person diagnosis by physician/trained personnel using reference standard required	Newcastle–Ottawa Scale; mean 8.3/9	Identified 33 predisposing and 112 precipitating factors; substantial physiologic heterogeneity precluded simple universal weighting.	NA	Broad risk-factor evidence partially overlaps frailty-specific R09 [41] and perioperative guideline evidence.	Not POD-only; no single pooled effect for most factors; broad populations/settings.
R07	[35] C. J. Swarbrick and J. S. L. Partridge (2022) DOI: 10.1111/anae.15607	Narrative review	NR (narrative review)	NR	Strategies to reduce POD incidence in older surgical patients	Risk assessment, nonpharmacologic and perioperative strategies	Varied across cited studies	No formal systematic risk-of-bias/GRADE process reported for the narrative review	Emphasizes multicomponent prevention and individualized perioperative management; several proposed strategies remain context-dependent.	Discussed qualitatively	Broad overlap with R02/R08/R13/R14 and many primary trials.	Narrative selection/interpretive bias; not exhaustive.
R08	[38] C. Aldecoa et al. (2017) DOI: 10.1097/EJA.0000000000000594	Evidence- and consensus-based clinical guideline	Through Mar 2015 (pre-update evidence base)	Multiple evidence reviews; aggregate count NR	Adult surgical patients; POD prevention/detection/management	Perioperative risk reduction, monitoring, drugs, nonpharmacologic care	Validated delirium assessment emphasized	Evidence grading + expert consensus	Established perioperative POD recommendations later updated by R02 [8].	Safety incorporated into recommendations.	Substantially superseded by R02 [8]; overlaps R13/R14/R18/R19.	Older evidence base; use mainly for continuity/historical guideline concordance.
R09	[41] H. Wu, S. Yan, H. Cao, C. Feng, and H. Zhang (2025) DOI: 10.1186/s12871-025-02994-3	Systematic review + meta-analysis	Through 29 Jun 2024	43 cohort studies; 14,441 patients	Older surgical patients; preoperative physical frailty and POD	Frailty exposure	International/validated delirium assessment required by eligibility	Newcastle–Ottawa Scale	Frailty associated with POD: pooled OR 2.47 (95% CI 2.04–2.99), I²=46.7%.	NA	Frailty subset overlaps some risk-factor cohorts summarized in R06 [34].	Observational cohorts; heterogeneity in frailty/POD instruments and surgery types.
R10	[42] F. Chen, Z.-X. Wu, Q. Chen, D.-K. Zuo, X.-M. Ye, and H. Li (2026) DOI: 10.1136/bmjopen-2025-102444	Systematic review + meta-analysis	Inception–Mar 2024	12 observational studies (9 POD, 3 POCD)	Surgical populations with blood/CSF NfL measurement	NfL as biomarker/exposure	POD definitions/instruments varied by included study	Newcastle–Ottawa Scale; PRISMA; sensitivity/publication-bias analyses	Preop blood NfL associated with POD in 5-study pool: OR 1.75 (95% CI 1.01–3.01), I²=73.9%; postoperative/between-group NfL differences also observed.	NA (biomarker studies)	Biomarker evidence partly overlaps broad narrative R07 and mechanistic review R11.	Observational evidence; high heterogeneity; assay/timing variability; no validated clinical threshold.
R11	[43] Z. Chen et al. (2023) DOI: 10.3389/fnins.2023.1145318	Narrative/mechanistic review	NR	NR	Tau-related mechanisms and anesthetic-associated cognitive disorders	Tau biology/anesthetic mechanisms	Not a POD-specific systematic ascertainment synthesis	No formal systematic appraisal reported	Supports biological plausibility of tau-related neuronal vulnerability, not a validated diagnostic or treatment threshold.	NA	Mechanistic overlap with biomarker sections in R07/R10.	Indirect for POD; much evidence mechanistic/preclinical or broader cognitive outcomes.
R12	[47] H. Feng et al. (2024) DOI: 10.1007/s12035-023-03864-0	Narrative review	NR	NR	Perioperative exercise and perioperative neurocognitive disorders	Exercise/prehabilitation	Mixed PND outcomes; POD not consistently primary	No formal systematic appraisal reported	Physical function/exercise plausibly affects perioperative resilience, but delirium-specific clinical evidence is heterogeneous and limited.	Exercise feasibility/safety discussed qualitatively	Overlaps primary prehabilitation RCTs P03–P06.	Narrative; includes preclinical and broader cognition outcomes.

ID	Ref.	Review / evidence-synthesis type	Final search date	No. studies/participants	Population coverage	Intervention/exposure/comparator	Delirium ascertainment coverage	Appraisal method	Principal finding used in manuscript	Harms/safety	Important overlap with other included reviews	Key limitations/directness
R13	[49] S. K. Inouye et al. (2015) DOI: 10.1016/j.jamcollsurg.2014.10.019	AGS best-practice statement/guideline	Comprehensive searches 1988–Dec 2013 for prevention/treatment; other topics based on high-quality guidelines/reviews	6,504 records identified in comprehensive search; selected evidence tables by topic	Older surgical adults	Risk factors, screening, prevention, treatment	Validated screening/diagnostic instruments; daily postoperative screening may be considered	Evidence tables + quality ratings; multidisciplinary 23-member expert panel and consensus	Recommends multicomponent nonpharmacologic prevention and avoidance of deliriogenic medications; pharmacologic therapy limited to selected circumstances.	Medication harms and safety explicitly considered.	Substantial overlap with R08/R02/R14 and older multicomponent evidence including P14/R19.	Older evidence base; some topics rely on prior reviews/guidelines.
R14	[50] C. G. Hughes et al. (2020) DOI: 10.1213/ANE.0000000000004641	Consensus statement with structured literature review	Search period reported within consensus methods; final date NR in manuscript extraction	163 studies met inclusion criteria	Adult perioperative POD prevention	Systems, risk stratification, anesthetic/medication/nonpharmacologic prevention	Validated POD tools across contributing literature	Formal consensus recommendations with level-of-evidence framework	Recommends multidisciplinary/system-level prevention and avoidance of overreliance on a single intervention.	Safety/feasibility included in recommendations.	Overlaps R13/R08 and later R02; primary studies also represented in later systematic reviews.	Consensus statement; evidence quality varies across questions.
R15	[51] A. L. Meulenbroek, S. R. Van Mil, M. C. Faes, F. U. S. Mattace-Raso, I. Fourneau, and L. Van Der Laan (2022) DOI: 10.1016/j.avsg.2022.04.005	Systematic review	Last run 21 Oct 2021	4 studies; 565 patients	Vascular surgery	Delirium-prevention strategies	Varied across included studies	Cochrane RoB for RCTs; ROBINS-I for observational studies	Very limited vascular-specific evidence; some benefit signals for geriatric assessment/prehabilitation but insufficient for broad extrapolation.	Sparse reporting	Population-specific subset of broader prevention evidence in R02/R07/R14/R18.	Only 4 studies; heterogeneous interventions; small evidence base.
R16	[57] L. Huang and Y. Zhang (2023) DOI: 10.3389/fmed.2023.1280013	Systematic review + meta-analysis	NR in extraction source	15 studies reported in review; meta-analytic subsets varied	Older adults undergoing noncardiac surgery	Intravenous vs inhalational general anesthesia	Postoperative cognition outcomes; POD not consistently isolated	Systematic-review methods; quality/risk-of-bias assessment reported by authors	Cognitive outcomes compared across anesthetic techniques; not definitive POD-specific evidence.	Broader anesthesia outcomes variably reported	Potential trial overlap with broad TIVA review R17; endpoints differ.	Indirect for POD because cognitive-test outcomes and delirium are not interchangeable.
R17	[58] N. Daccache et al. (2025) DOI: 10.1016/j.bja.2025.02.007	Systematic review + meta-analysis of RCTs	Inception–10 Oct 2024	385 RCTs; 55,223 participants (>55,000)	Adult and pediatric; predominantly noncardiac surgery; TIVA vs inhalational anesthesia	TIVA (TCI/manual) vs inhalational anesthesia	Broad safety/recovery outcomes; POD not a principal universal outcome	Cochrane risk-of-bias assessment; certainty grading reported	Comparable major adverse-event safety; TIVA reduced PONV/emergence agitation; no universal POD-preventive advantage established.	ClassIntra grade 3–4 adverse events RR 1.00 (95% CI 0.88–1.12); PONV favored TIVA.	Potential overlap with R16 and some anesthesia RCTs; much broader outcome scope.	POD-specific evidence indirect; mixed ages/surgeries; 125 RCTs high overall RoB.
R18	[60] J. K. Burton et al. (2021) DOI: 10.1002/14651858.CD013307.pub3	Cochrane systematic review + meta-analysis	Through 16 Sep 2020	22 RCTs; 5,718 participants; 14 multicomponent trials (3,693)	Hospitalized adults outside ICU/HDU; medical and surgical populations	Nonpharmacological delirium-prevention interventions	Delirium incidence required; instruments varied by trial	Cochrane risk-of-bias tool + GRADE; component NMA exploratory	Multicomponent interventions RR 0.57 (95% CI 0.46–0.71), I^2=39%; moderate-certainty evidence.	Little/no clear mortality effect; nonpharmacologic interventions generally low direct harm but resource-intensive.	Substantial overlap with older trials in R19 [62], including HELP-derived interventions and some surgical prevention studies.	Non-ICU; intervention components heterogeneous; individual component effects difficult to isolate.
R19	[62] T. T. Hshieh et al. (2015) DOI: 10.1001/jamainternmed.2014.7779	Systematic review + meta-analysis	1 Jan 1999–31 Dec 2013	14 articles; 12 unique trials; ~4,267 patients	Acute medical and surgical wards	Multicomponent nonpharmacological delirium prevention	Validated delirium assessment required	Two independent reviewers; Cochrane risk-of-bias scoring	Overall odds of delirium lower with multicomponent care: OR 0.47 (95% CI 0.38–0.58); NNT ≈14.3.	No major intervention harm signal; resource intensity not captured by effect estimate	Includes HELP/HELP-adapted trials; substantial overlap with evidence later incorporated in R18 [60].	Mix of randomized and nonrandomized designs; older literature; heterogeneity in components.

ID	Ref.	Review / evidence-synthesis type	Final search date	No. studies/participants	Population coverage	Intervention/exposure/comparator	Delirium ascertainment coverage	Appraisal method	Principal finding used in manuscript	Harms/safety	Important overlap with other included reviews	Key limitations/directness
R20	[63] By the 2023 American Geriatrics Society Beers Criteria® Update Expert Panel (2023) DOI: 10.1111/jgs.18372	Expert consensus criteria update with evidence review	2017–2022 literature for 2023 update	Topic-specific evidence tables; aggregate count NR	Adults ≥65 y across ambulatory, acute and institutional settings (except hospice/end-of-life)	Potentially inappropriate medications/medication safety	Not a POD-specific effectiveness review	GRADE-informed evidence review; ACP framework; AMSTAR considerations for reviews; expert panel consensus	Identifies anticholinergic, sedative and other medications with potentially unfavorable risk-benefit profiles in older adults.	Medication adverse effects central to criteria	Medication-safety evidence complements POD-specific guidance R02/R08/R13 and observational P02.	Not POD-specific; criteria require individualization and should not be applied mechanically.
R21	[64] D. Fang, L. Zeng, and F. Kuang (2025) DOI: 10.1186/s12912-025-03013-z	Systematic review + meta-analysis	Inception–1 Dec 2024	18 RCTs; 2,717 ICU patients	ICU delirium prevention/treatment care bundles	Care bundles vs standard care	Delirium outcomes across RCTs; instruments/bundle composition varied	Two reviewers; Cochrane Quality Assessment Tool	Delirium incidence RR 0.38 (95% CI 0.32–0.45); duration WMD −1.60 d (95% CI −1.96 to −1.23); substantial heterogeneity/bundle variability.	No mortality difference: RR 0.78 (95% CI 0.44–1.40); bundle safety generally contextual	Conceptually overlaps nonpharmacologic bundle reviews R18/R19 but ICU-specific interventions/populations differ.	ICU, not POD-specific; many studies moderate/high RoB; heterogeneous bundles; illness-severity confounding across implementation.
R22	[65] D. Wang, X. He, Z. Li, H. Tao, and C. Bi (2024) DOI: 10.1186/s12871-024-02453-5	Systematic review + meta-analysis of RCTs	Through 1 Jun 2023	18 RCTs (10 POD-focused, 8 broader POCD outcomes)	Older patients undergoing regional anesthesia	IV dexmedetomidine adjunct	POD instruments/timing varied across trials	Cochrane-style risk-of-bias assessment	Pooled POD signal favored dexmedetomidine (RR ≈0.48, 95% CI 0.37–0.63 in POD subset).	Bradycardia/hypotension remain relevant adverse effects	Overlaps some dexmedetomidine trials in broader NMA R05; different anesthesia context from cardiac R04.	Regional-anesthesia subset; mixed regimens and outcomes; some trials small.
R23	[68] M. Hallschmid (2021) DOI: 10.1111/jne.12934	Narrative review	NR	NR	Intranasal insulin across metabolic/cognitive neuroscience; largely chronic cognitive impairment rather than perioperative POD	Intranasal insulin	Not a POD-specific review	No formal systematic risk-of-bias appraisal reported	Central insulin signaling can be pharmacologically modulated, but effects vary by phenotype, dose, device and disease context.	Hypoglycemia is generally minimized by intranasal delivery but systemic/other adverse effects and device issues remain relevant.	Mechanistic/clinical background overlapping the intranasal-insulin node in R05 [32].	Mostly non-perioperative and non-POD evidence; indirect for clinical implementation.
R24	[69] A. K. Alwesabi et al. (2026) DOI: 10.3389/fendo.2026.1745202	Narrative review	NR	NR	Diabetes, POD and longer-term postoperative cognitive dysfunction in adults	Metabolic vulnerability / diabetes-related mechanisms	Mixed delirium and cognitive outcomes	No formal systematic appraisal reported	Diabetes/metabolic dysregulation may increase vulnerability, but observational associations do not validate cerebral metabolic therapies.	NA	Mechanistic overlap with R23 and general vulnerability reviews.	Narrative; mixed outcomes; cannot infer benefit of correcting peripheral glycemia for cerebral insulin signaling/POD prevention.
R25	[14] X. Ding, M. Zhang, J. Liu, and D. Wu (2026) DOI: 10.3389/fnins.2026.1759910	Narrative review	NR	NR	Older surgical patients; POD mechanisms, prediction, prevention/treatment	Broad perioperative POD strategies	Varied cited-study instruments	No formal systematic appraisal reported	Broad contemporary overview of pathophysiology, risk prediction and therapeutic strategies.	Discussed qualitatively	Broad overlap with R26–R29 and with primary evidence mapped elsewhere in S2.	Narrative review; overlap is conceptual and study-level independence cannot be assumed.
R26	[15] A. Smirnov, M. Semionov, V. Yasinski, Y. Binyamin, A. Zlotnik, and D. Frank (2025) DOI: 10.3390/ijms262311314	Narrative review	NR	NR	Postoperative delirium in elderly patients	Broad mechanisms/risk/prevention	Varied	No formal systematic appraisal reported	Contemporary narrative overview of POD in older adults.	Qualitative	Broad conceptual overlap with R25/R28/R29 and guideline evidence.	Narrative; no study-level overlap quantification.

ID	Ref.	Review / evidence-synthesis type	Final search date	No. studies/participants	Population coverage	Intervention/exposure/comparator	Delirium ascertainment coverage	Appraisal method	Principal finding used in manuscript	Harms/safety	Important overlap with other included reviews	Key limitations/directness
R27	[16] O. K. Dilmen, B. C. Meco, L. A. Evered, and F. M. Radtke (2024) DOI: 10.1016/j.jclinane.2023.111320	Clinical narrative guide	NR	NR	Postoperative neurocognitive disorders; vulnerable surgical patients	Safe Brain / perioperative risk-reduction approach	Distinguishes delirium from other PND phenotypes	No formal systematic appraisal reported	Clinically oriented guide emphasizing vulnerability recognition and individualized perioperative brain-health strategies.	Qualitative	Conceptual overlap with R02/R07/R14 and R28/R29.	Broader PND focus; not a systematic POD effect synthesis.
R28	[17] S. Ahmadzadeh et al. (2025) DOI: 10.7759/cureus.92927	Narrative review	NR	NR	Postoperative delirium clinical management	Detection, multicomponent prevention, pharmacologic strategies	Varied	No formal systematic appraisal reported	Synthesizes current POD management and highlights unresolved questions in hemodynamics, EEG and biomarkers.	Qualitative	Broad overlap with R02/R07/R25/R29 and mapped primary trials.	Narrative; no independent pooled estimate.
R29	[18] D. S. Paternò et al. (2026) DOI: 10.3390/jcm15031253	Narrative review	NR	NR	Perioperative neurocognitive disorders; mechanisms, prevention, management, biomarkers	Broad PND strategies	Mixed PND/POD	No formal systematic appraisal reported	Integrates neuroinflammation, perfusion, neurotransmitters, vulnerability, prevention and biomarkers.	Qualitative	Broad conceptual overlap with R25–R28 and multiple evidence rows.	PND umbrella; narrative; not all cognitive outcomes are POD.
R30	[3] E. R. Marcantonio (2017) DOI: 10.1056/NEJMcp1605501	Clinical review	NR	NR	Delirium in hospitalized older adults	Diagnosis, prognosis and management	Clinical diagnosis / validated tools discussed	Narrative clinical review	Foundational clinical overview, including hypoactive delirium and adverse outcomes.	Qualitative	Background overlap with delirium consensus/guidelines.	Not perioperative-specific.
R31	[10] D. Meagher (2009) DOI: 10.1080/09540260802675460	Narrative review of motor subtypes	NR	NR	Motor subtypes of delirium across settings	Phenotyping rather than intervention	Motor subtype definitions vary across literature	Narrative review	Describes hyperactive, hypoactive and mixed delirium motor phenotypes and prognostic variation.	NA	Phenotype background overlaps general delirium reviews.	Older, nonsurgical/mixed literature; not an intervention synthesis.

S2C. Claim-to-evidence traceability map

Target ID	Table 3 classification	Evidence-map row(s)
**T3-01**	Multicomponent non-pharmacological prevention (HELP-based principles) — routine practice	R02, R08, R18, R19; P14
**T3-02**	Structured delirium surveillance linked to a clinical response pathway — routine practice	P01, R03
**T3-03**	Medication review/reduction of deliriogenic exposure — routine practice	P02, R02, R08, R20
**T3-04**	Early mobilization, sensory optimization, hydration/nutrition, sleep protection, and adequate analgesia — routine bundle components	R02, R08, R18, R19; P14
**T3-05**	ABCDEF / ICU liberation principles — selected contexts	R21
**T3-06**	Processed EEG-guided anesthetic titration — selected contexts	P07, P08, P09, P10, P11
**T3-07**	Cerebral oximetry (NIRS) — selected contexts	R02, R07, R08
**T3-08**	Dexmedetomidine — selected contexts	R04, R05, R22
**T3-09**	Melatonin / melatonin-receptor agonists — selected contexts	R02, R05, R07, R08
**T3-10**	Antipsychotics — selected rescue treatment, not routine prophylaxis or disease modification	P15, P16, R02, R08
**T3-11**	Prehabilitation — investigational for POD prevention	P03, P04, P05, P06; R12
**T3-12**	Blood-based biomarker-guided risk stratification — investigational	R06, R07, R10, R11
**T3-13**	Intranasal insulin / cerebral metabolic targeting — investigational	R02, R05, R07, R08, R23
