# Supplementary material for: Postoperative Delirium Across the Perioperative Pathway: A Structured Narrative Review of Clinical Maturity and Implementation Priorities
Source: Diagnostics (Basel). 2026 Oct 27;16(21):3518. doi: 10.3390/diagnostics16213518 (PMC13762076; doi:10.3390/diagnostics16213518)
Supplement: Supplementary file 1 [file diagnostics-16-03518-s001.zip › Supplementary Figure S1.pdf]

Supplementary Figure S1. PRISMA-style flow diagram illustrating identification and selection of literature for the structured narrative review.

IDENTIFICATION

**Records identified from bibliographic databases (n = 26348)**<br>PubMed/MEDLINE (n =5964)<br>Web of Science (n = 7511)<br>CENTRAL (n =3675)<br>Scopus (n =9198)	**Records identified from register (n = 313)**<br>ClinicalTrials.gov (n = 313)<br>**Records/reports identified from other sources (n = 29)**<br>ESAIC / ASA resources (n = 2)<br>Pre-2021 foundational sources considered separately (n = 27)
↓	↓
**Records removed before screening**<br>Duplicate records removed before screening: n = 12270<br>Records screened after deduplication: n = 14078	**Other-source records/reports assessed separately**<br>Not retained after initial assessment (n = 314)

SCREENING

**Unique database records screened by title/abstract (n = 14078)**	**Records excluded at title/abstract stage (n = 11038)**

↓

Reports sought for full-text retrieval (n = 3040)

INCLUDED IN THE STRUCTURED NARRATIVE SYNTHESIS

Records captured by the main database searches (n=32)
Records identified by supplementary targeted database/reference searching (n=10)
Records/reports identified from other sources (n = 28)

↓ ↓

Total publications informing the structured narrative synthesis (n = 70)
